# Perforin Expression Directly *Ex Vivo* by HIV-Specific CD8^+^ T-Cells Is a Correlate of HIV Elite Control

**DOI:** 10.1371/journal.ppat.1000917

**Published:** 2010-05-27

**Authors:** Adam R. Hersperger, Florencia Pereyra, Martha Nason, Korey Demers, Prameet Sheth, Lucy Y. Shin, Colin M. Kovacs, Benigno Rodriguez, Scott F. Sieg, Leia Teixeira-Johnson, Debbie Gudonis, Paul A. Goepfert, Michael M. Lederman, Ian Frank, George Makedonas, Rupert Kaul, Bruce D. Walker, Michael R. Betts

**Affiliations:** 1 Department of Microbiology, University of Pennsylvania, Philadelphia, Pennsylvania, United States of America; 2 Ragon Institute of MGH, MIT, and Harvard, Boston, Massachusetts, United States of America; 3 Biostatistics Research Branch, National Institutes of Health, Bethesda, Maryland, United States of America; 4 Department of Medicine, University of Toronto, Toronto, Ontario, Canada; 5 Canadian Immunodeficiency Research Collaborative, Toronto, Ontario, Canada; 6 Case Western Reserve University and University Hospitals of Cleveland, Cleveland, Ohio, United States of America; 7 Department of Infectious Diseases, Cleveland Clinic, Cleveland, Ohio, United States of America; 8 Division of Infectious Diseases, Department of Medicine, University of Pennsylvania, Philadelphia, Pennsylvania, United States of America; 9 Department of Medicine, University of Alabama at Birmingham, Birmingham, Alabama, United States of America; 10 Howard Hughes Medical Institute, Chevy Chase, Maryland, United States of America; Johns Hopkins School of Medicine, United States of America

## Abstract

Many immune correlates of CD8^+^ T-cell-mediated control of HIV replication, including polyfunctionality, proliferative ability, and inhibitory receptor expression, have been discovered. However, no functional correlates using *ex vivo* cells have been identified with the known ability to cause the direct elimination of HIV-infected cells. We have recently discovered the ability of human CD8^+^ T-cells to rapidly upregulate perforin—an essential molecule for cell-mediated cytotoxicity—following antigen-specific stimulation. Here, we examined perforin expression capability in a large cross-sectional cohort of chronically HIV-infected individuals with varying levels of viral load: elite controllers (n = 35), viremic controllers (n = 29), chronic progressors (n = 27), and viremic nonprogressors (n = 6). Using polychromatic flow cytometry and standard intracellular cytokine staining assays, we measured perforin upregulation, cytokine production, and degranulation following stimulation with overlapping peptide pools encompassing all proteins of HIV. We observed that HIV-specific CD8^+^ T-cells from elite controllers consistently display an enhanced ability to express perforin directly *ex vivo* compared to all other groups. This ability is not restricted to protective HLA-B haplotypes, does not require proliferation or the addition of exogenous factors, is not restored by HAART, and primarily originates from effector CD8^+^ T-cells with otherwise limited functional capability. Notably, we found an inverse relationship between HIV-specific perforin expression and viral load. Thus, the capability of HIV-specific CD8^+^ T-cells to rapidly express perforin defines a novel correlate of control in HIV infection.

## Author Summary

While the majority HIV-infected individuals progress to AIDS, a fraction of these individuals—for reasons not completely understood—do not develop AIDS and also display sustained control over viral replication; these subjects are sometimes referred to as elite controllers (EC). Prior evidence has shown that HIV-specific CD8^+^ T-cells, a component of adaptive immunity against intracellular pathogens, from EC exhibit enhanced functionality compared to individuals with progressive disease. Therefore, HIV-specific CD8^+^ T-cells likely play an important role in the favorable clinical outcomes witnessed in EC. We show in this study that the ability to control HIV replication in EC is associated with the expression of a protein called perforin, a critical molecule that enables CD8^+^ T-cells to directly kill infected cells - thereby preventing the spread of HIV to previously uninfected cells. In infected subjects with nonprogressive disease, we show that HIV-specific CD8^+^ T-cells demonstrate a superior ability to express perforin upon antigen-specific stimulation, whereas in progressors this property is diminished. Thus, we identify a functional capability of CD8^+^ T-cells, readily measured by standard intracellular cytokine staining assays, that potentially has a direct impact on HIV replication *in vivo*. These findings may, therefore, provide an important qualifier for future HIV vaccine research.

## Introduction

Approximately 35–40 million people are currently infected with HIV worldwide. Most of these individuals fail to control HIV replication, and ultimately progress to acquired immune deficiency syndrome (AIDS) if left untreated. However, a subset (<1%) of the HIV-infected population, termed elite controllers (EC), can spontaneously control viral replication to undetectable levels [1], [2], [3]. Understanding the mechanisms of immunologic control of HIV replication in EC may identify candidate markers of immune control useful for assessing HIV vaccine strategies.

The host immune response, in particular HIV-specific CD8^+^ T-cells, is at least partially responsible for the control of viral replication in many EC. For example, EC are enriched for certain HLA alleles, such as HLA-B13, B15, B51, B27, B57, and B58 [4], [5], [6], [7]. EC contain a greater fraction of HIV-specific CD8^+^ T-cells that can degranulate, produce multiple functional cytokines and chemokines and display markedly better proliferative potential upon HIV peptide stimulation than individuals with progressive disease [7], [8], [9], [10], [11], [12], [13]. Additionally, recent evidence has demonstrated that HIV-specific CD8^+^ T-cells from EC have enhanced cytotoxic capabilities compared to progressors: Several groups have shown that HIV-specific CD8^+^ T-cells from EC display a superior ability to suppress the replication of HIV during extended culture [14], [15], [16]. Using CD8^+^ T-cells expanded *in vitro* for six days, Migueles and colleagues observed a higher cytotoxic capacity on a per-cell basis of HIV-specific CD8^+^ T-cells from EC [17]. Collectively, these findings suggest that CD8^+^ T-cells may be critical to the control of HIV replication *in vivo*.

CD8^+^ T-cells are thought to kill virally-infected cells predominantly through the release of lytic proteins - mainly perforin and granzymes - that are secreted via exocytosis of pre-formed granules following recognition of infected targets [18], [19], [20]. Granule-mediated killing by CD8^+^ T-cells occurs within minutes to hours of target cell recognition; however, the reconstitution of intracellular perforin following degranulation has been reported to first require cellular proliferation [13], [21], [22]. We have recently identified another mechanism by which perforin-mediated CD8^+^ T-cell killing can take place: the rapid upregulation and targeted release of newly produced perforin, which traffics to the immunological synapse via a route that largely bypasses cytotoxic granules [23]. *De novo* synthesis of perforin by human CD8^+^ T-cells can be detected by flow cytometry in conjunction with standard intracellular cytokine-staining (ICS) [24], thus permitting simultaneous assessment of CD8^+^ T-cell cytotoxic potential and cytokine production.

Here, we measured the ability of HIV-specific CD8^+^ T-cells to express perforin in a cross-sectional cohort of chronically-infected individuals that differentially control viral replication. Several previously published studies have examined perforin expression in HIV-specific CD8^+^ T-cells in both progressive and nonprogressive infection [13], [25], [26], [27]. However, due to the nature of the anti-perforin antibody employed [23], these studies have uniformly assessed only pre-formed, granule-associated perforin present within resting or long-term activated HIV-specific CD8^+^ T-cells. In this work we demonstrate that HIV-specific CD8^+^ T-cells from EC, compared to progressors, have a superior ability to express perforin immediately upon activation, without the need for prior proliferation or the addition of exogenous cytokines. Overall, this work identifies the rapid expression of perforin as a novel correlate of control of HIV replication and urges a closer examination of CD8^+^ T-cell polyfunctionality in HIV infection.

## Results

### HIV-specific CD8^+^ T-cell response between EC and CP did not vary greatly in total magnitude, degranulation, or cytokine production

We assessed the magnitude and functional characteristics of HIV-specific CD8^+^ T-cells by stimulating PBMC from 35 elite controllers (EC), 29 viremic controllers (VC), and 27 chronic progressors (CP) [Table 1 and Table S1] with overlapping peptide pools encompassing all HIV-1 (clade B) proteins. We developed a flow cytometric staining panel (Fig. S1A) that simultaneously measured memory phenotype (CD27, CD45RO, and CD57), degranulation [surface expression of CD107a [28]], cytokine expression (IFN-γ, TNFα, and IL-2), and chemokine production (MIP1α). As a sixth functional parameter, we included an anti-perforin antibody (clone B-D48) to measure perforin upregulation [23], [24]. As shown in Figure S1B, the historically used antibody (δG9 clone) cannot detect perforin expression within activated CD8^+^ T-cells in the same ICS assay format.

**Table 1:** Clinical parameters of HIV infected subject cohorts.

Patient Characteristics	Elite Controller	Viremic Controller	Chronic Progressor	Viremic Nonprogressor	HAART-treated
Number of subjects	35	29	27	6	15
Plasma HIV RNA, **median** (*IQR*), copies/mL	undetectable	**396** (*82*–*874*)	**24,121** (*18,000*–*41,579*)	**35,000** (*29,672*–*101,500*)	undetectable
CD4^+^ T-cell count, **median** (*IQR*), cells/mm^3^	**811** (*702*–*1,068*)	**576** (*449*–*785*)	**508** (*401*–*599*)	**557** (*439*–*625*)	**440** (*301*–*610*)
Decline in CD4^+^ T-cell count per year, **median** (*IQR*), cells/mm^3^	Not determined	Not determined	**170** (*103*–*319*)	**36** (*26*–*47*)	Not determined
Infection duration, **median** (*IQR*), years	**17** (*13*–*21*)	**12** (*8*–*19*)	**7** (*4*–*13*)	**20** (*16*–*22*)	**16** (*12*–*20*)
Duration of HAART treatment prior to PBMC sample, **median** (*IQR*), years	N/A	N/A	N/A	N/A	**2** (*1*–*6*)

**Figure 1:**
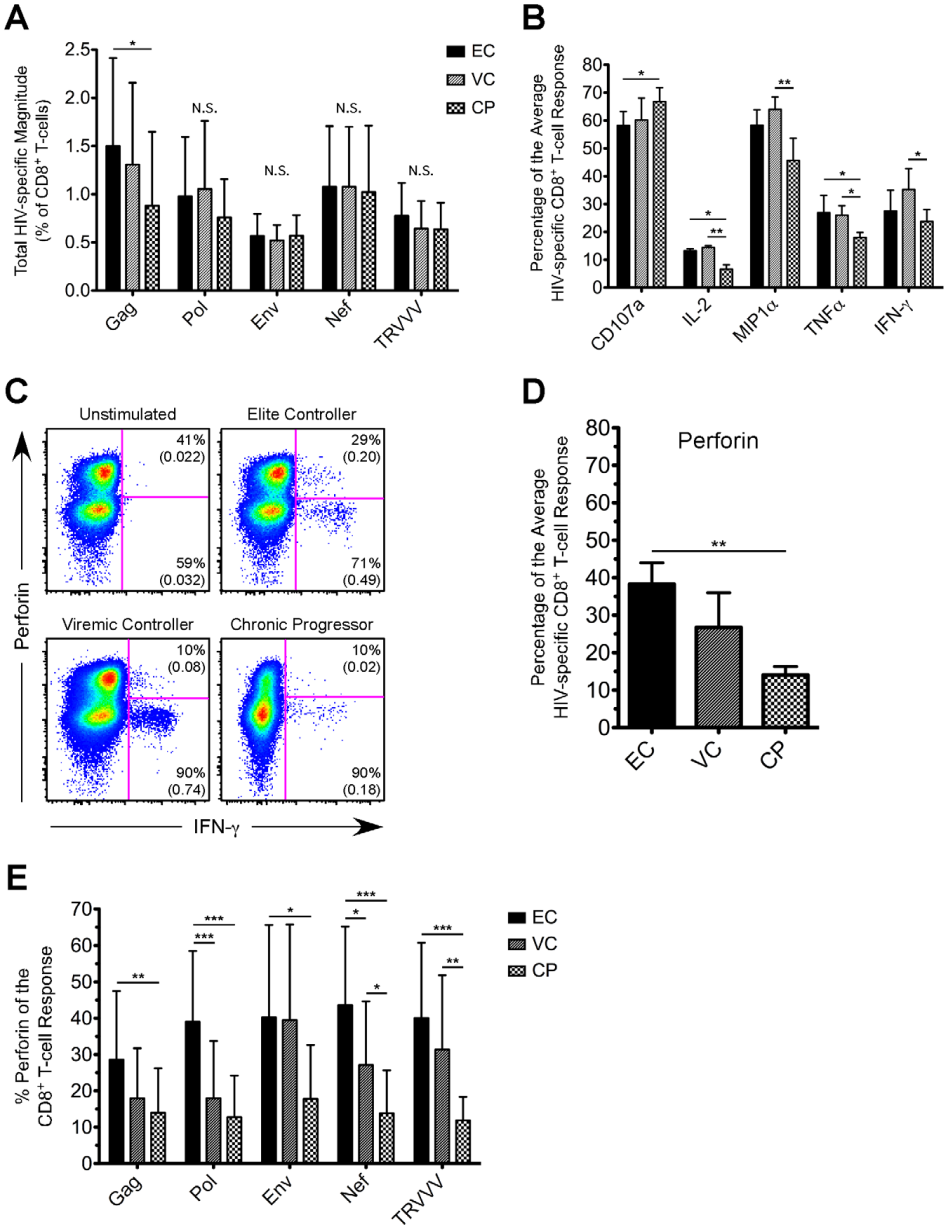
HIV-specific CD8^+^ T-cells in EC demonstrate an enhanced ability to express perforin compared to CP. (A) The CD8^+^ T-cell response magnitude to all HIV peptide pools was calculated for EC, VC, and CP and plotted as percent of CD8^+^ T-cells (excluding naïve cells). The total magnitude was calculated by summing across all functional combinations. (A) The proportion of the average HIV-specific CD8^+^ T-cell response comprised of each single functional parameter (except perforin) is shown for EC, VC, and CP. (C) Representative flow cytometric plots of perforin versus IFN-γ are shown from one representative EC, VC, and CP. Percentages represent the proportion of functional cells that stain either positive or negative for perforin. Values in parentheses are the magnitude of each population and denote percent of CD8^+^ T-cells (excluding naïve cells). All reported values have been corrected for background. (D) The proportion of the average HIV-specific CD8^+^ T-cell response comprised of perforin is shown for EC, VC, and CP. (E) The relative contribution of perforin to the Gag-, Pol-, Env-, Nef-, and TRVVV-specific CD8^+^ T-cell responses is shown for EC, VC, and CP. (A, B, D, E) Statistical analysis was carried out using one-way ANOVA tests (nonparametric; Kruskal-Wallis) followed by a Dunns test for multiple comparisons. * denotes a p value <0.05, ** denotes a p value<0.01, and *** denotes a p value <0.001. All bars represent the mean and error bars indicate the standard deviation.

As shown in Figure 1A, the total HIV-specific CD8^+^ T-cell response magnitude to Pol, Env, Nef, or TRVVV stimulation did not differ substantively across the groups, but EC displayed a somewhat higher Gag-specific response. The lack of large differences in response magnitude is in agreement with previous studies that measured the total magnitude of CD8^+^ T-cell responses in EC and CP using flow cytometry [7], [8], [13]. We next determined the relative contribution of CD107a, IFN-γ, TNFα, IL-2, and MIP1α to the HIV-specific CD8^+^ T-cell response (Fig. 1B). In general, no clear trends emerged in overall functionality between the groups. For example, compared to EC, CP demonstrated a slightly enhanced ability to degranulate, lower levels of TNFα, but no statistically significant difference in the proportion of the average HIV-specific CD8^+^ T-cell response comprised of either IFN-γ or MIP1α. The largest difference in functionality was IL-2 expression, which was higher among EC and VC compared to CP. Previous studies have also shown enhanced production of IL-2 after HIV-specific stimulation in subjects with low or undetectable viremia [7], [8], [10]. Similar overall observations were found for the individual HIV antigens as well (data not shown).

### HIV-specific CD8^+^ T-cells in EC demonstrated a greater ability to express perforin than VC and CP

We next assessed perforin expression by HIV-specific CD8^+^ T-cells in each cohort group. We consistently observed higher co-expression of perforin within responding cells from EC compared to VC or CP for all HIV antigens (Fig. 1C shows representative Nef-specific responses producing IFN-γ; other HIV antigens are not shown but yielded similar results). In fact, perforin expression comprised a significantly greater proportion of the average HIV-specific CD8^+^ T-cell response in EC than in CP (Fig. 1D). The relative contribution of perforin to the CD8^+^ T-cell response was significantly higher (∼3 fold) in EC compared to CP for all of the individual HIV antigens (Fig. 1E). In addition to the proportion of the HIV-specific CD8^+^ T-cell response comprised of perforin, EC also displayed a greater magnitude of perforin expression upon stimulation by all HIV antigen pools compared to both VC and CP (Fig. S2). However, we found no correlation among EC between the total magnitude of an HIV-specific response and the corresponding amount of perforin expression (Fig. S3).

As shown in Figure S4, there was, however, some variability among EC subjects in the contribution of perforin to the HIV-specific CD8^+^ T-cell response. Within some EC there was low perforin expression induced by one HIV antigen (e.g. Gag) but higher perforin production to another peptide pool (e.g. Pol). Some EC demonstrated high HIV-specific perforin in response to every antigen. Although several EC did express low levels of HIV-specific perforin, only 20% of all EC in the cohort failed to achieve 30% perforin for the CD8^+^ T-cell response to at least one of the antigen pools (data not shown). In contrast, only 15% of CP demonstrated even one HIV antigen-specific CD8^+^ T-cell response comprised of 30% perforin (data not shown). Thus, our data suggests that EC are not simply a homogenous group of HIV-infected individuals and do demonstrate some variability, which is in agreement with previous findings [7], [29].

**Figure 2:**
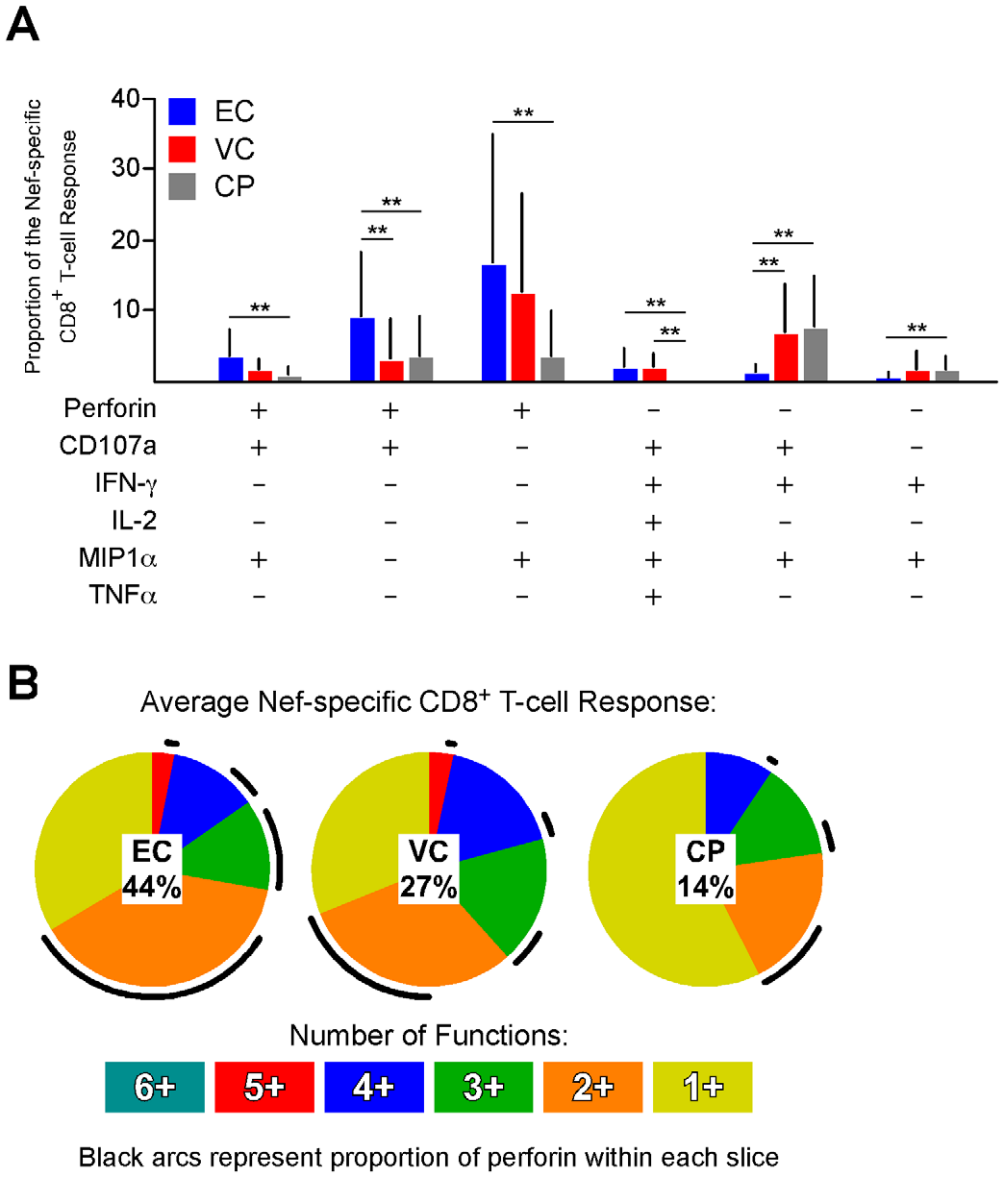
The majority of perforin expression comes from cells with otherwise limited functional capability. (A) The functionality of the average Nef-specific CD8^+^ T-cell response is shown; only the functional permutations that varied significantly between at least two of the groups are shown. ** denotes a p value <0.01 based on a Lachenbruch's Two-part Wilcoxon test as described in the Methods. All bars represent the mean and error bars indicate the standard deviation. (B) The average Nef-specific CD8^+^ T-cell functional profile is shown for EC, VC, and CP. Responses are grouped according to the number of positive functions. The relative amount of perforin positivity within each functional group (i.e. each pie slice) is depicted as black arcs. The relative contribution of perforin (mean value) to the entire response is represented by the percentage in the center of each pie.

Next, we examined the functional profile of the average Nef-specific CD8^+^ T-cell response among the EC, VC, and CP groups (Fig. 2A and 2B; the other HIV antigens are not shown but yielded similar results). Only the functional combinations that were significantly different between at least two of the groups are shown in Figure 2A; all 64 combinations are shown in Figure S5. We rarely observed simultaneous expression of all six functions because perforin and IL-2 are generally not co-expressed by the same cell [30]. The average Nef-specific functional profile in EC and VC was composed of more CD8^+^ T-cells than in CP that simultaneously expressed five functions (Fig. 2B). Additionally, the percentage of the Nef-specific response that was perforin-positive (black arcs in Fig. 2B) was significantly higher among EC (44%) compared to VC (27%; p<0.05) or CP (14%; p<0.001). Similar findings were observed for Gag-, Pol-, Env-, and TRVVV-specific responses (data not shown).

The majority of perforin was produced by cells expressing only a single other function: CD107a or MIP1α (Fig. 2B). The CD8^+^ T-cells that co-expressed CD107a and perforin likely upregulated perforin *de novo* since a cell that was CD107a^+^ presumably lost all (or nearly all) of its granule-associated perforin through the process of degranulation. As shown in Figure S6, the proportion of the HIV-specific response in EC that was both CD107a^+^ and perforin^+^ was significantly higher than CP for all HIV antigens. The second major population of perforin^+^ cells co-expressed only MIP1α. The relevance of this population is unclear. However, we have previously shown that activated CD8^+^ T-cells can transport newly synthesized perforin directly to the immunological synapse without trafficking first through cytolytic granules [23]. Thus, despite the absence of apparent degranulation, MIP1α^+^ perforin^+^ cells may potentially be involved in ongoing cytolytic activity.

### Perforin expression is not restricted to the presence of protective HLA-B alleles

**Figure 3:**
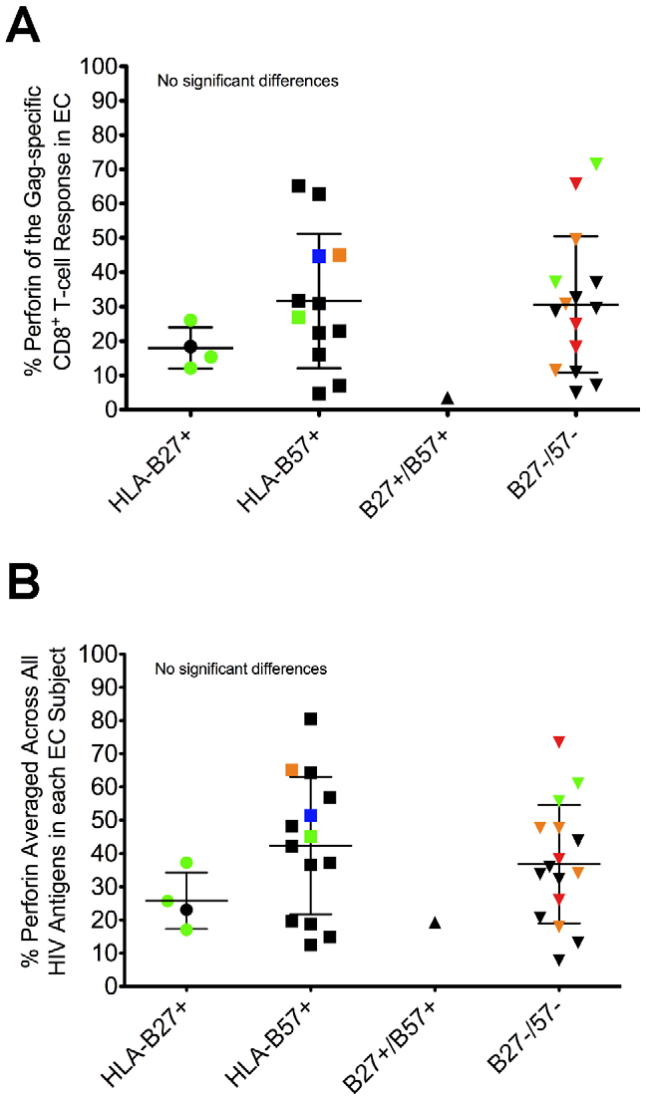
Perforin expression is not restricted to the presence of protective HLA-B alleles. EC were stratified based on the expression of HLA-B alleles previously shown to be associated with improved clinical outcomes. The relative amount of perforin expression is shown for the (A) Gag-specific and (B) average HIV-specific CD8^+^ T-cell responses among all EC. Each symbol represents an individual study subject. Some of the symbols are colored to denote the presence of another protective HLA-B allele: blue, HLA-B13; green, HLA-B15; orange, HLA-B51; red, HLA-B58. No statistically significant differences were found between the groups using a one-way ANOVA test (nonparametric; Kruskal-Wallis) followed by a Dunns test for multiple comparisons. The error bars represent the mean and standard deviation.

One consistent host factor associated with durable control of HIV is the presence of certain HLA class I alleles, particularly HLA-B27 and B57 [1], [4], [31], [32], [33]. Other HLA-B alleles have also been associated with delayed disease progression or lower viral loads, including HLA-B13, B15, B51, and B58 [5], [6]. Among EC in our cohort, 54% of the subjects expressed HLA-B27 or B57, while 32% of VC carried these alleles (data not shown). Additionally, 43% of EC in the study cohort expressed either HLA-B13, B15, B51, or B58, while 32% of VC carried these alleles (data not shown). As shown in Figures 3A and S7, we found no association between protective HLA-B status and perforin expression to any HIV peptide pool in either EC or VC (Gag shown in Fig. 3A, Nef shown in Fig. S7, and data not shown), or when perforin expression to all HIV peptide pools was averaged within each subject (Fig. 3B). Thus, there was no apparent relationship between protective HLA-B alleles and the capacity of HIV-specific CD8^+^ T-cells to express perforin after stimulation.

### Distinct expansion of HIV-specific effector CD8^+^ T-cells in EC

**Figure 4:**
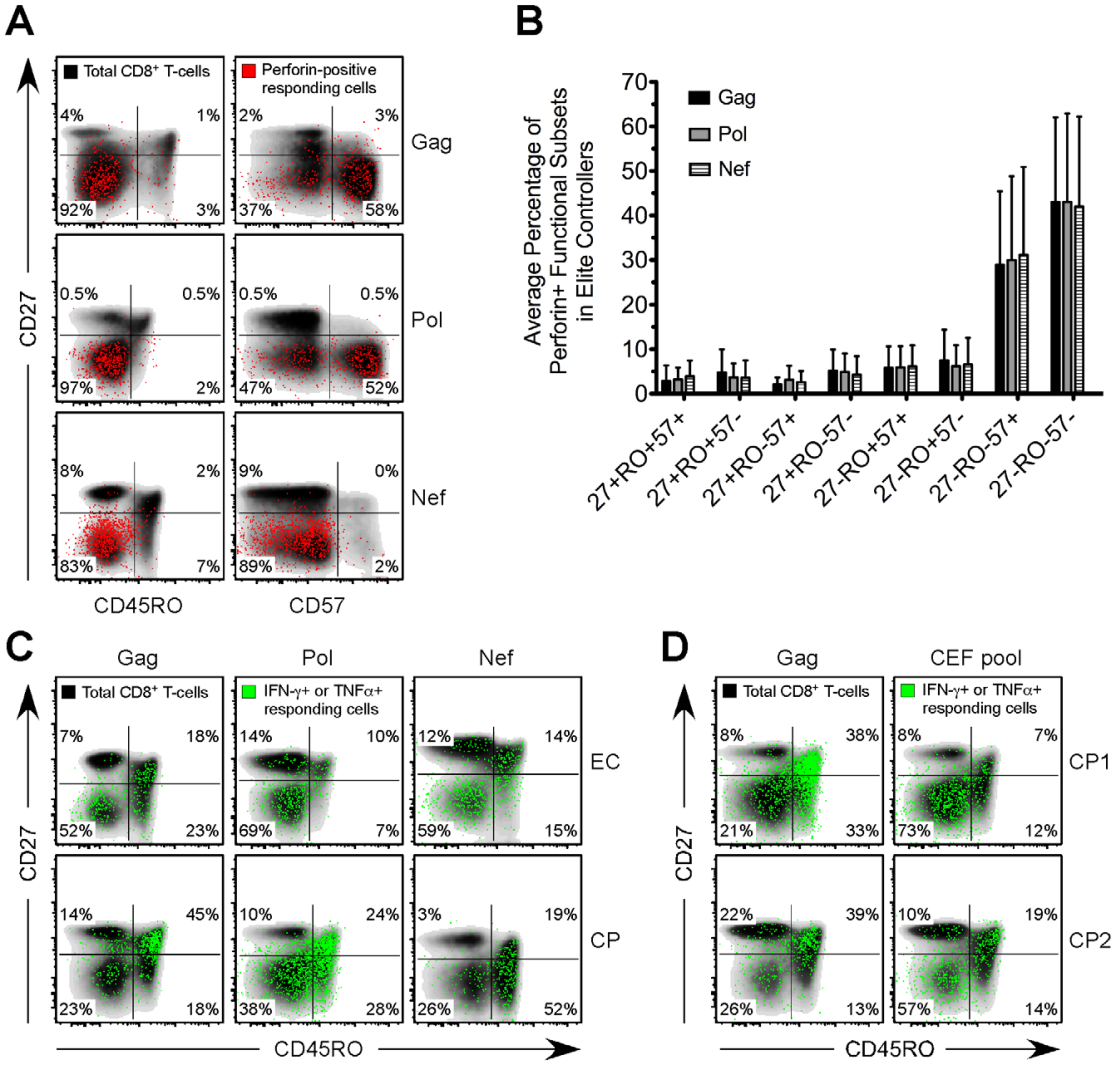
Distinct expansion of HIV-specific effector CD8^+^ T-cells in EC. (A) Gag-, Pol-, and Nef-specific perforin^+^ functional subsets (red events) were overlaid onto a density plot (black shading) of the memory phenotype, as determined by CD27, CD45RO, and CD57, of the total CD8^+^ T-cell population in three representative EC subjects. (B) The memory phenotype of Gag-, Pol-, and Nef-specific perforin^+^ functional subsets, as determined by CD27, CD45RO, and CD57, was determined for all EC. Bars represent the mean and error bars indicate the standard deviation. (C) Gag-, Pol-, and Nef-specific CD8^+^ T-cells, as defined by the production of IFN-γ or TNFα (green events), were overlaid onto a density plot (black shading) of the memory phenotype, as determined by CD27 and CD45RO, of the total CD8^+^ T-cell population in three separate EC and CP subjects. (D) Gag- and CEF-specific CD8^+^ T-cells, as defined by the production of IFN-γ or TNFα (green events), were overlaid onto a density plot (black shading) of the memory phenotype, as determined by CD27 and CD45RO, of the total CD8^+^ T-cell population in two separate CP subjects. (A, C, D) Percentages represent the fraction of overlaid cells that fall within each quadrant.

We next examined the memory phenotype, based on surface expression of CD27, CD45RO, and CD57, of HIV-specific CD8^+^ T-cells in each group. The majority of HIV-specific CD8^+^ T-cells that expressed perforin in EC, VC, and CP were CD27^-^CD45RO^-^CD57^+/-^ (Fig. 4A and 4B and data not shown), commonly considered an effector-type profile, which is in agreement with previous reports that examined the presence of perforin in various human CD8^+^ T-cell memory subsets [34], [35]. This phenotype was common to virtually all perforin^+^ HIV-specific CD8^+^ T-cells regardless of their specificity for Gag, Pol, Nef, Env or TRVVV (Fig. 4A and data not shown). HIV-specific CD8^+^ T-cells among many CP subjects were skewed toward a CD27^+^CD45RO^+/-^ memory phenotype (Fig. 4C), as previously shown [8], [36], [37]. However, a higher proportion of HIV-specific CD8^+^ T-cells in EC than in CP displayed a memory phenotype consistent with highly differentiated effector cells (Fig. 4C). Overall, the presence of CD27^-^CD45RO^-^ HIV-specific CD8^+^ T-cells was less common among CP than EC (Fig. S8) in agreement with a previous study [14]. The absence of effector-like HIV-specific CD8^+^ T-cells in CP is not, however, reflective of the total CD8^+^ T-cell pool in these individuals. A substantial fraction of CD8^+^ T-cells in CP that responded to CEF stimulation were CD27^-^CD45RO^-^ (Fig. 4D). However, responding Gag-specific CD8^+^ T-cells within the same subjects were primarily CD27^+^CD45RO^+^ (Fig. 4D).

### Inverse relationship between HIV-specific perforin expression and viral load

**Figure 5:**
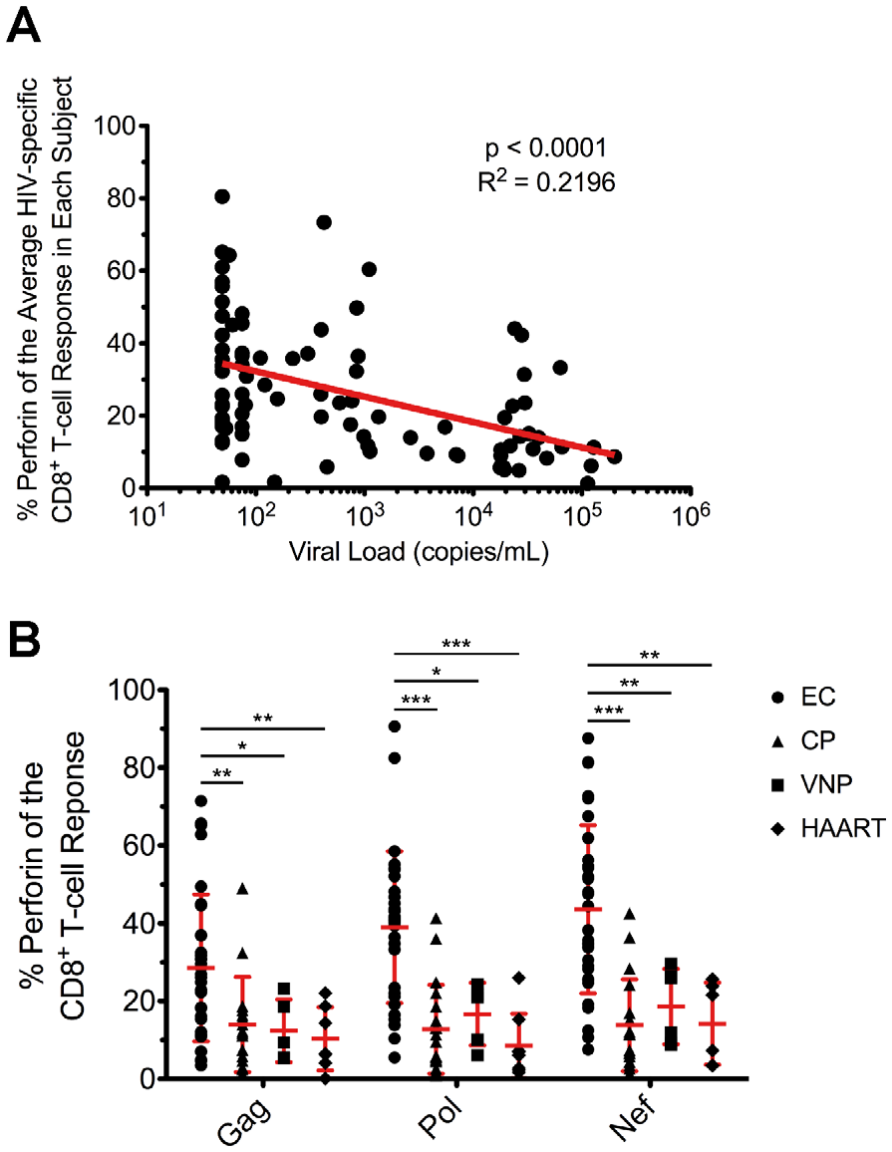
Inverse relationship between viral load and HIV-specific perforin expression, which is not rescued by HAART. (A) The average proportion of HIV-specific perforin expression within each subject was plotted against the HIV viral load from each respective subject. The most proximal viral load measurement to the time point of the PBMC sample was used in the analysis. Spearman correlation tests (nonparametric; two-tailed) were performed to determine statistical significance. (B) The relative contribution of perforin to the Gag-, Pol-, and Nef-specific CD8^+^ T-cell responses is shown for all EC, CP, VNP, and HAART-suppressed subjects. Each symbol represents an individual study subject. One-way ANOVA tests (nonparametric; Kruskal-Wallis test) were performed followed by a Dunns test for multiple comparisons. * denotes a p value <0.05, ** denotes a p value <0.01, and *** denotes a p value <0.001. The error bars represent the mean and standard deviation.

Having observed higher perforin expression in HIV-specific CD8^+^ T-cells in EC, we next examined the relationship between perforin expression and viral load. We found a significant inverse correlation between the average HIV-specific perforin expression within each subject and HIV viral load (Fig. 5A). This inverse relationship was found for every individual HIV antigen specificity (data not shown) and when only considering subjects with detectable viremia (EC subjects excluded; Fig. S9). We also found a statistically significant positive correlation between CD4^+^ T-cell counts in the blood and HIV-specific perforin expression by CD8^+^ T-cells across all subjects (Fig. S10), a finding most likely driven by the high CD4^+^ T-cell counts among the EC subjects (Table 1). Furthermore, when we examined the other functional parameters in a similar manner, we only found a statistically significant inverse relationship between IL-2 expression and viral load (Fig. S11), which is an expected result based upon previous studies.

To better understand the relationship between viral load and perforin expression, we next compared HIV-specific CD8^+^ T-cell responses among EC to viremic nonprogressors (VNP), who maintain stable CD4^+^ T-cell counts in the face of consistently high viral loads (median 35,000 viral RNA copies/mL plasma; Table 1) without progressing to AIDS. The infection duration in both groups was similar (17 vs. 20 years in the absence of therapy; Table 1). Therefore, by comparing these two groups, we can control for the rate of CD4^+^ T-cell decline, progression rate, and duration of infection. As shown in Figure 5B, perforin expression by HIV-specific CD8^+^ T-cells in VNP is significantly lower than EC and actually closely resembles the perforin levels observed in CP. Together, these data indicate that the degree of HIV-specific CD8^+^ T-cell perforin expression is predictive of the ability to control viral load independent of the rate of CD4^+^ T-cell decline, progression status, or infection duration.

### HIV-specific perforin expression is not recovered by HAART

In order to determine whether the low perforin expression associated with progression was reversible, we examined HIV-specific perforin expression by CD8^+^ T-cells in HAART-treated individuals with undetectable HIV viremia (Table 1). Compared to EC, the total CD8^+^ T cell response magnitude was lower in HAART-treated subjects to Gag, Pol, and Nef stimulation; however, only the difference in the total Gag-specific magnitude reached statistical significance (Fig. S12). Despite some differences in total magnitude, there were no substantive differences in the relative contribution of degranulation, IFN-γ, TNFα, or MIP1α production (Fig. S13). However, HIV-specific perforin expression in HAART-suppressed subjects was considerably lower than EC, and was similar to the levels observed in CP (Fig. 5B). Thus, the ability to express and rapidly upregulate perforin by HIV-specific CD8^+^ T-cells in chronic HIV infection is not recovered following HAART.

## Discussion

While many cell surface markers, activation profiles, and functional parameters of both *ex vivo* HIV-specific CD8^+^ and CD4^+^ T-cells have been shown to correlate with control of viremia [8], [38], [39], [40], [41], [42], few, if any, can potentially mediate direct control of HIV replication through the lysis of infected cells. Here we have shown that perforin expression by *ex vivo* HIV-specific CD8^+^ T-cells is significantly higher in EC compared to patients with uncontrolled viral replication. HIV-specific CD8^+^ T-cells that express perforin bear predominantly an effector phenotype, indicating that effector populations, in addition to central memory populations [43], [44], may be critically important to the control of HIV infection. We also find an inverse correlation between perforin expression by HIV-specific CD8^+^ T-cells and viral load. Together, these results represent an unique assessment of HIV-specific immunity and provide a novel platform for measuring potential vaccine efficacy in clinical trials.

There is little question regarding the crucial importance of perforin in the control of infectious pathogens. Indeed, mutation or dysregulation of perforin in humans results in compromised cellular immunity and enhanced susceptibility to viral infections [45]. Previous reports on *ex vivo* HIV-specific CD8^+^ T-cells have uniformly found low or absent perforin expression in both CP and EC and no detectable differences in perforin levels between the groups [13], [25], [27], [37], [46]. However, these studies have in retrospect only defined the level of granule-associated perforin within resting HIV-specific CD8^+^ T-cells due to unforeseen limitations in the anti-perforin antibody employed in these studies [23], [24]. Due to chronic activation and continual presence of viral antigen - albeit extremely low levels in EC [47] - HIV-specific CD8^+^ T-cells are unlikely to reach a true resting state; therefore, it is unlikely these cells accumulate cytolytic granules containing perforin *in vivo*. However, our results indicate that this does not preclude their ability to upregulate new perforin upon antigen-specific stimulation, a killing mechanism that we have recently shown potentiates the cytotoxic ability of human CD8^+^ T-cells [23].

We have shown previously that both the commonly used anti-perforin antibody (δG9 clone) and the anti-perforin antibody used in this study (B-D48 clone) stain resting CD8^+^ T-cells equivalently [24]. Thus, previous research that found no difference in the levels of perforin within resting HIV-specific CD8^+^ T-cells between EC and CP [13], [25], [27], [37], [46] were not necessarily incorrect. Here, we have shown using a perforin antibody that can detect both granule-associated and granule-independent forms of perforin that HIV-specific CD8^+^ T-cells from EC express this protein to a higher degree than patients with uncontrolled viremia. It is important to note, though, that the B-D48 clone cannot specifically distinguish pre-formed from newly upregulated perforin using flow cytometric-based assays. Nevertheless, to identify the potential contribution of perforin produced *de novo*, we examined the proportion of the HIV-specific CD8^+^ T-cell response that both degranulated (CD107a^+^) yet remained perforin^+^ after six hours of stimulation. These CD8^+^ T-cells that co-expressed CD107a and perforin likely upregulated new perforin; they have presumably lost most or all of their pre-formed perforin through the process of degranulation. By analyzing this specific population, we found that the proportion of the HIV-specific CD8^+^ T-cell response in EC that co-expressed CD107a^+^ and perforin^+^ was significantly higher than CP to all HIV antigens. However, we have also shown that newly synthesized perforin largely bypasses cytotoxic granules [23]; therefore, we are almost certainly underestimating the levels of perforin upregulation by focusing only on cells that have degranulated.

The capacity of unstimulated CD8^+^ T-cells from EC to begin to eliminate HIV-infected autologous CD4^+^ T-cell targets within several hours of co-incubation has been previously reported [14]. The results from this study suggested that HIV-specific CD8^+^ T-cells were responsible for the elimination of infected CD4^+^ T-cells through a mechanism dependent on cell-to-cell contact and MHC-I restriction. Our findings on perforin upregulation by HIV-specific CD8^+^ T-cells shortly after stimulation are consistent with the results of Saez-Cirion and colleagues [14] and may even be a mechanism to explain their findings. Moreover, another previously published report indicated that HIV-specific CD8^+^ T-cells kill targets through the use of cytotoxic granules and not by the Fas/FasL pathway [48]. Therefore, available evidence indicates that the perforin/granzyme pathway of cytotoxicity is likely the primary means by which HIV-specific CD8^+^ T-cells kill infected cells *in vivo*.

Our findings here suggest that HIV-specific CD8^+^ T-cells in EC have a superior cytotoxic potential by expressing higher levels of perforin. This supposition is supported by recent work from Migueles and colleagues [17]. These authors showed that HIV-specific CD8^+^ T-cells from EC accumulate more granule-associated perforin as a result of their superior ability to proliferate *in vitro* compared to CD8^+^ T-cells from progressors. They also found that higher amounts of perforin (and granzyme B) in HIV-specific cells translate into an enhanced ability to lyse infected targets. Thus, together with the previous work of Migueles et al., our results show that EC have an enhanced ability to upregulate perforin either directly *ex vivo* or after *in vitro* proliferation. Given what is known about the importance of perforin in orchestrating cytotoxicity, we can conclude that HIV-specific CD8^+^ T-cells from EC certainly have the potential to elicit elimination of infected targets to a greater degree than progressors, which may directly impact viral load. Furthermore, we know that newly synthesized perforin traffics directly to the immunological synapse - the site of action of cytotoxicity [23].

Besides differences in cytotoxic capabilities, HIV-specific CD8^+^ T-cells from EC have also been shown to be more polyfunctional in nature; they can simultaneously degranulate and produce multiple functional molecules, such as IL-2, IFN-γ, and TNFα, to a greater extent than CD8^+^ T-cells from progressors [8], [49]. Our results here confirm and extend these findings. Polyfunctional HIV-specific CD8^+^ T-cells were also found in this study to comprise a greater fraction of the response in EC than in CP. Interestingly, we rarely observed HIV-specific CD8^+^ T-cells capable of producing all six functions simultaneously. This results from a dichotomous relationship between perforin and IL-2 production from the same cell [30]. The implications of this dichotomy are profound for our understanding of effective HIV-specific CD8^+^ T-cell responses: IL-2 producing CD8^+^ T-cells will presumably not have immediate cytolytic activity; conversely, perforin producing CD8^+^ T-cells may be inherently reliant upon production of IL-2 from cells in their surrounding environment for maintenance or modulation. Both cell types are most likely crucial to maintaining protective immunity. The IL-2 producing cells may be part of a population of CD8^+^ T-cells that can maintain itself through autocrine production of IL-2. This ability may be important in the setting of diminished CD4^+^ T-cell help in HIV infection [10]. Alternatively, these cells may represent a self-renewing memory population of CD8^+^ T-cells responsible for long-term maintenance of effector cells. IL-2 producing cells likely do not display any direct anti-viral capability directly after activation [30] but may be able to differentiate into perforin producing effector cells. The increased IL-2 production observed by both HIV-specific CD8^+^ T-cells and CD4^+^ T-cells [42] in EC may also directly increase cytotoxic potential, as has recently been reported [50], [51].

Interestingly, we found that a substantial fraction of the total perforin production by HIV-specific CD8^+^ T-cells among EC comes not from polyfunctional populations but instead from cells that elicit only a single other measured functional parameter: specifically MIP1α or CD107a. In previous studies, where perforin upregulation was not measured, the potential importance and cytotoxic capabilities of these populations was not appreciated. On this note, the degree of functionality of a CD8^+^ T-cell response is only reflective of what functional parameters are actually being measured. For example, we find that most CD8^+^ T-cells that upregulate perforin also produce granzyme B upon stimulation [30]. Therefore, many of the CD8^+^ T-cells found in this study to co-express perforin with MIP1α and/or CD107a, may actually be highly “polyfunctional” if we had also examined the expression of other parameters critical for cytotoxicity, such as granzyme B.

Our data show that perforin expressing cells bear effector-like phenotypic markers. Thus, while a central memory phenotype is often considered a protective phenotype in HIV infected individuals, our results suggest that effector cells are also of significance. It should be noted, however, that simply achieving effector status does not guarantee the expression of perforin. Indeed, some HIV-specific CD8^+^ T-cells in both EC and CP were CD27^-^CD45RO^-^ yet did not express perforin. Our results suggest that effector status is necessary but not sufficient for perforin upregulation. The importance of effector cells in the control of HIV infection is further supported by recent observations by Picker and colleagues who found that a rhesus-CMV-based SIV vaccine vector could stimulate protective effector SIV-specific CD8^+^ T-cells [52].

Our results suggest that perforin expression by HIV-specific CD8^+^ T-cells is not readily recovered by inhibition of viral replication or reduction in chronic immune activation by HAART - a finding which is consistent with a previous report showing that HAART treatment does not restore other functional parameters, such as proliferative capacity, polyfunctionality, or cytotoxic capacity [53]. We also found that perforin production does not appear to be directly influenced by beneficial HLA-B haplotypes or the relative maintenance of CD4^+^ T-cell levels over time. Whether perforin expression is lost early, late, or progressively during infection remains unclear. Further studies are necessary to identify the mechanism(s) underlying the relative absence of perforin upregulation in progressive HIV infection, and, if possible, to discover a means by which this critical function can be regained or elicited through therapeutic intervention.

## Materials and Methods

### Ethics statement

Blood specimens were acquired with the written informed consent of all study patients and with the approval of the institutional review board at each respective institution where patient materials were collected: University of Pennsylvania (IRB# 809316), University Hospitals Case Western Medical Center (IRB# FWA00003937), University of Alabama at Birmingham (IRB# X090708004), University of Toronto and St. Michael's Hospital (IRB# 07-106), and Harvard University (IRB# 2003-P-001894 and IRB# 2003-P-001678/75). The study was conducted following the principles stipulated in the Declaration of Helsinki.

### Human subjects

We examined *ex vivo* HIV-specific CD8^+^ T-cell responses from 35 elite controllers (EC), 29 viremic controllers (VC), 27 chronic progressors (CP), and 6 viremic nonprogressors (VNP). Most EC and VC were recruited from outpatient clinics at local Boston hospitals as well as from providers throughout the United States [54]. Several EC were also recruited from clinics associated with the University of Toronto. PBMC samples from CP were from clinics associated with the University of Pennsylvania Center for AIDS Research, the University of Toronto, Case Western Reserve University, and the University of Alabama at Birmingham. VNP samples were obtained from the University of Toronto and Case Western Reserve University. PBMC samples from 15 HAART-suppressed patients were obtained from Harvard University and the University of Toronto.

EC were defined by consistent plasma HIV RNA levels below the limit of detection (e.g. <75 copies/mL by bDNA or <50 copies/mL by ultrasensitive PCR) in a minimum of three determinations of plasma HIV RNA spanning at least a 12-month period. VC consistently maintained viral load between 50 and 2,000 copies/mL, while the majority of viral load measurements of CP were above 10,000 copies/mL. CD4^+^ T-cell counts were not considered for inclusion criteria in the EC, VC, or CP groups. VNP were identified as subjects with consistently high viremia (above 10,000 copies/mL on average) but with relatively stable CD4^+^ T-cell counts after long-term infection. It is the relative preservation of CD4^+^ T-cell numbers in spite of sustained high level HIV replication that was used to distinguish the VNP group clinically from CP. All subjects from the EC, VC, CP, and VNP groups were off antiretroviral therapy for at least 6 months prior to the sampling date; yet most subjects were treatment-naive. Refer to Table 1 and Table S1 for more detailed information on the study cohort.

### Antibodies

The following antibodies were used in this study: anti-CD4 PE Cy5.5, anti-CD14 APC Alexa 750, anti-CD19 APC Alexa 750, anti-CD8 Texas Red-PE, anti-IFN-γ Alexa 700 (Invitrogen, Carlsbad, CA), anti-CD107a FITC, anti-IL-2 APC, anti-TNFα PE Cy7 (BD Pharmingen, San Diego, CA), anti-MIP1α PE (R&D Systems, Minneapolis, MN), anti-CD27 PE Cy5 (Beckman Coulter, Fullerton, CA), anti-CD57 Qdot 565, anti-CD3 Qdot 585, and anti-CD45RO Qdot 605 or 705 (custom). Custom conjugations to Quantum (Q) dot nanocrystals were performed in our laboratory with reagents purchased from Invitrogen. The anti-perforin antibody (B-D48 clone) was purchased from Diaclone (Besancon, France) and conjugated to Pacific Blue (Invitrogen) in our laboratory.

### PBMC stimulation assays

Cryopreserved PBMC were thawed and subsequently rested overnight at 37°C, 5% CO_2_ in complete medium (RPMI supplemented with 10% FBS and 1% L-glutamine). The following morning, the cells were washed with complete medium and resuspended at a concentration of 2×10^6^ cells/mL if sufficient cell numbers were available. Costimulatory antibodies (anti-CD28 and anti-CD48d; each at 1 µg/ml final concentration; BD Biosciences; San Jose, California), monensin (1 µg/ml final concentration; BD Biosciences; San Jose, California) and Brefeldin A (1 µg/ml final concentration; Sigma-Aldrich; St. Louis, Missouri) were also added to each condition. Anti-CD107a was added at the start of all stimulation periods, as described previously [28]. PBMC were incubated at 37°C, 5% CO_2_ for six hours with overlapping 15-mer peptide pools encompassing HIV-1 (clade B) Gag, Pol, Env, Nef, and the viral accessory proteins (TRVVV) [as 5 separate conditions]. PBMC from many of the subjects were also stimulated with a CEF peptide pool, which contains peptides derived from CMV, EBV, and Influenza virus. Each individual peptide in the pools was at a final concentration of 2 µg/mL for all stimulations.

At the end of six hours, cells were stained with Aqua amine-reactive dye (Invitrogen; Carlsbad, California) for 15 minutes in the dark at room temperature in order to later identify viable cells. A cocktail of antibodies was then added to the cells to stain for surface markers for an additional 20 minutes. Following staining for cell surface molecules, cells were permeabilized using the Cytofix/Cytoperm kit (BD Biosciences; San Jose, California) according to the manufacturer's instructions. A cocktail of antibodies against intracellular markers was then added to the cells and allowed to incubate for one hour in the dark at room temperature. Finally, cells were fixed in 1x PBS containing 1% paraformaldehyde (Sigma-Aldrich; St. Louis, Missouri) before being stored in the dark at 4°C until the time of collection on the flow cytometer.

### Flow cytometric analysis

For each stimulation condition, at least 500,000 total events were acquired using a modified LSRII (BD Immunocytometry Systems, San Jose, California). Data analysis was performed using FlowJo (version 8.8.4; TreeStar, Ashland, Oregon) and Spice (version 4.2.3, Dr. Mario Roederer, NIH, Bethesda, Maryland). Reported data have been corrected for background, and only responses with a total frequency above 0.25% of memory CD8^+^ T-cells (after background subtraction) were considered for analysis. Boolean gating analysis was carried out once positive gates were established for each functional parameter. This analysis resulted in 64 possible combinations of the 6 measured functions. Importantly, two combinations were ignored in all analyses: (1) events negative for all measured functional parameters and (2) perforin single-positive cells. By analyzing the data in such a manner, we only examined perforin expression resulting from HIV-specific stimulation. For this reason, perforin expression was only considered within activated, HIV-specific CD8^+^ T-cells expressing at least one other functional parameter. Refer to Figure S1A for further information on the gating strategy. As indicated by the gating strategy, naïve cells (CD27^+^CD45RO^-^) were excluded when performing all analyses except for the memory phenotyping data presented in Figure 4.

### Statistical analysis

All graphing and statistical analysis was performed using R (version 2.8.1), JMP (version 7), or GraphPad Prism software (version 5.0a). Functionality was compared between study groups using nonparametric tests (Mann-Whitney test for two groups; Kruskal-Wallis test followed by a Dunns test for multiple comparisons when comparing three or more groups). Correlations between viral load or CD4^+^ T-cell counts and perforin expression were based on Spearman correlation coefficients. Comparisons between groups of specific functional permutations were based on a Lachenbruch's Two-part Wilcoxon test. This analysis simultaneously tests for a difference in the proportion of subjects who have an above zero response and a difference in the magnitude of the response [55], [56]. Only those functional combinations for which the average response was greater than zero were considered to be relevant for consideration. Functional permutations were considered significantly different if the p value was below 0.01. In all figures, * denotes a p value <0.05, ** denotes a p value <0.01, and *** denotes a p value <0.001. Unless otherwise noted, error bars represent the standard deviation.

## Supporting Information

Figure S1 **Perforin upregulation can be detected using a polychromatic flow cytometric staining panel.** (A) The gating strategy from a representative subject: PBMC from an EC were stimulated with SEB for six hours and then stained for six CD8^+^ T-cell functions (perforin, CD107a, IFN-γ, IL-2, TNFα, and MIP1α) along with lineage (CD14, CD19, CD3, CD4, CD8) and memory (CD27, CD45RO, and CD57) markers. The no stimulation control is also shown. (B) PBMC were stimulated with SEB for six hours in the presence of BFA and monensin. Perforin was stained either using the δG9 (*left*) or B-D48 (*right*) antibody clones. The red box denotes granule-associated perforin within the population of CD8^+^ T-cells that did not respond to SEB stimulation (i.e. resting CD8^+^ T-cells not producing IFN-γ). The black box denotes a population of CD8^+^ T-cells expressing both perforin and IFN-γ that can be detected using the B-D48 clone in a conventional ICS assay.
(0.60 MB TIF)

Figure S2 **EC demonstrate higher perforin magnitude than CP.** In addition to the relative contribution of perforin to the HIV-specific CD8^+^ T-cell response, EC also demonstrate higher perforin magnitude. Total perforin production induced by each HIV antigen pool is represented as the frequency of CD8^+^ T-cells (excluding naïve cells). One-way ANOVA tests (nonparametric; Kruskal-Wallis test) were performed followed by a Dunns test for multiple comparisons. * denotes a p value < 0.05, ** denotes a p value < 0.01, and *** denotes a p value < 0.001. All bars represent the mean and error bars indicate the standard deviation.
(0.08 MB TIF)

Figure S3 **There is no association between HIV response magnitude and corresponding perforin expression.** The Gag-, Pol-, and Nef-specific response magnitude (as the frequency of CD8^+^ T-cells; excluding naïve cells) is plotted against the corresponding proportion of perforin expression for each CD8^+^ T-cell response among all EC subjects. Spearman correlation tests (nonparametric; two-tailed) revealed no statistically significant relationship.
(0.16 MB TIF)

Figure S4 **EC demonstrate some variability in HIV-specific perforin expression.** The relative contribution of perforin for the CD8^+^ T-cell response to each HIV antigen pool is shown for a selected subset of EC. Each symbol represents a different EC subject, and symbols of the same color represent responses from the same individual. These subjects were chosen partly because they mounted a positive response to all five HIV peptide pools.
(0.06 MB TIF)

Figure S5 **Breakdown of the average Nef-specific response from EC, VC, and CP into all 64 possible functional permutations.** The entire response was broken down into the contribution of each functional combination for the average Nef-specific CD8^+^ T-cell response. Note that two functional permutations are ignored in the analysis: perforin single positive and all negative. All bars represent the mean and error bars indicate the standard deviation.
(0.14 MB TIF)

Figure S6 **EC have an increased capacity for** ***de novo*** **perforin synthesis.** The proportion of the CD8^+^ T-cell response comprised of every CD107a^+^perforin^+^ functional subset was calculated for all HIV antigens in EC, VC, and CP. One-way ANOVA tests (nonparametric; Kruskal-Wallis test) were performed followed by a Dunns test for multiple comparisons. * denotes a p value < 0.05, ** denotes a p value < 0.01, and *** denotes a p value < 0.001. All bars represent the mean and error bars indicate the standard deviation.
(0.10 MB TIF)

Figure S7 **Perforin expression is not restricted to the presence of protective HLA-B alleles.** EC were stratified based on the expression of HLA-B alleles previously shown to be associated with improved clinical outcomes. The relative amount of perforin expression is shown for the Nef-specific CD8^+^ T-cell responses among all EC. Each symbol represents an individual study subject. Some of the symbols are colored to denote the presence of another protective HLA-B allele: blue, HLA-B13; green, HLA-B15; orange, HLA-B51; red, HLA-B58. No statistically significant differences were found between the groups using a one-way ANOVA test (nonparametric; Kruskal-Wallis) followed by a Dunns test for multiple comparisons. The error bars represent the mean and standard deviation.
(0.21 MB TIF)

Figure S8 **EC display an expansion of CD27^-^CD45RO^-^ effector HIV-specific CD8^+^ T-cells.** The memory phenotype, based on the surface expression of CD27 and CD45RO, was determined for the average HIV-specific CD8^+^ T-cell response, as defined by the production of IFN-γ or TNFα, among EC and CP. Mann-Whitney tests (nonparametric; two-tailed) were performed for each phenotypic combination. ** denotes a p value < 0.01. All bars represent the mean and error bars indicate the standard deviation.
(0.09 MB TIF)

Figure S9 **Negative correlation between HIV-specific perforin expression and viral load when considering only VC, CP, and VNP subjects.** The average percentage of HIV-specific perforin expression from CD8^+^ T-cells within each subject was plotted against the HIV viral load among all subjects excluding EC. The most proximal viral load measurement to the time point of the PBMC sample was used in the analysis. Spearman correlation tests (nonparametric; two-tailed) were performed to determine statistical significance.
(0.09 MB TIF)

Figure S10 **Positive correlation between HIV-specific perforin expression and peripheral blood CD4^+^ T-cell counts.** The percentage of Gag-, Pol-, and Nef-specific perforin expression within each subject was plotted against CD4^+^ T-cell counts. The average percentage of HIV-specific perforin expression within each individual was also plotted against CD4^+^ T-cell counts. The most proximal CD4^+^ T-cell count to the time point of the PBMC sample was used in the analysis. Spearman correlation tests (nonparametric; two-tailed) were performed to determine statistical significance.
(0.17 MB TIF)

Figure S11 **Not all functional parameters are correlated with control of HIV replication.** The proportion of each measured functional parameter (except perforin) comprising the average HIV-specific CD8^+^ T-cell response in each subject was plotted against the HIV viral load from each respective subject. The most proximal viral load measurement to the time point of the PBMC sample was used in the analysis. Spearman correlation tests (nonparametric; two-tailed) were performed to determine statistical significance.
(0.31 MB TIF)

Figure S12 **HIV-specific CD8^+^ T-cell response magnitude is slightly higher in EC than HAART-treated individuals.** The CD8^+^ T-cell response magnitude to Gag, Pol, and Nef peptide pools was calculated for EC and HAART-treated subjects and plotted as percent of CD8^+^ T-cells (excluding naïve cells). The total magnitude was calculated by summing across all functional combinations. Mann-Whitney tests (nonparametric; two-tailed) were performed for each HIV antigen. ** denotes a p value < 0.01. All bars represent the mean and error bars indicate the standard deviation.
(0.06 MB TIF)

Figure S13 **The HIV-specific CD8^+^ T-cell response between EC and HAART-treated subjects does not vary greatly in degranulation, cytokine production, or chemokine expression.** The proportion of the average HIV-specific CD8^+^ T-cell response comprised of each single functional parameter (except perforin) is shown among EC and HAART-treated subjects. Mann-Whitney tests (nonparametric; two-tailed) were performed for each functional parameter. * denotes a p value < 0.05. All bars represent the mean and error bars indicate the standard deviation.
(0.09 MB TIF)

Table S1 **Complete study cohort with relevant clinical parameters.**
(1.50 MB TIF)
