# Supplementary figures and images for: Perforin Expression Directly Ex Vivo by HIV-Specific CD8+ T-Cells Is a Correlate of HIV Elite Control
Source: PLoS Pathog. 2010 May 27;6(5):e1000917. doi: 10.1371/journal.ppat.1000917 (PMC2877741; doi:10.1371/journal.ppat.1000917)

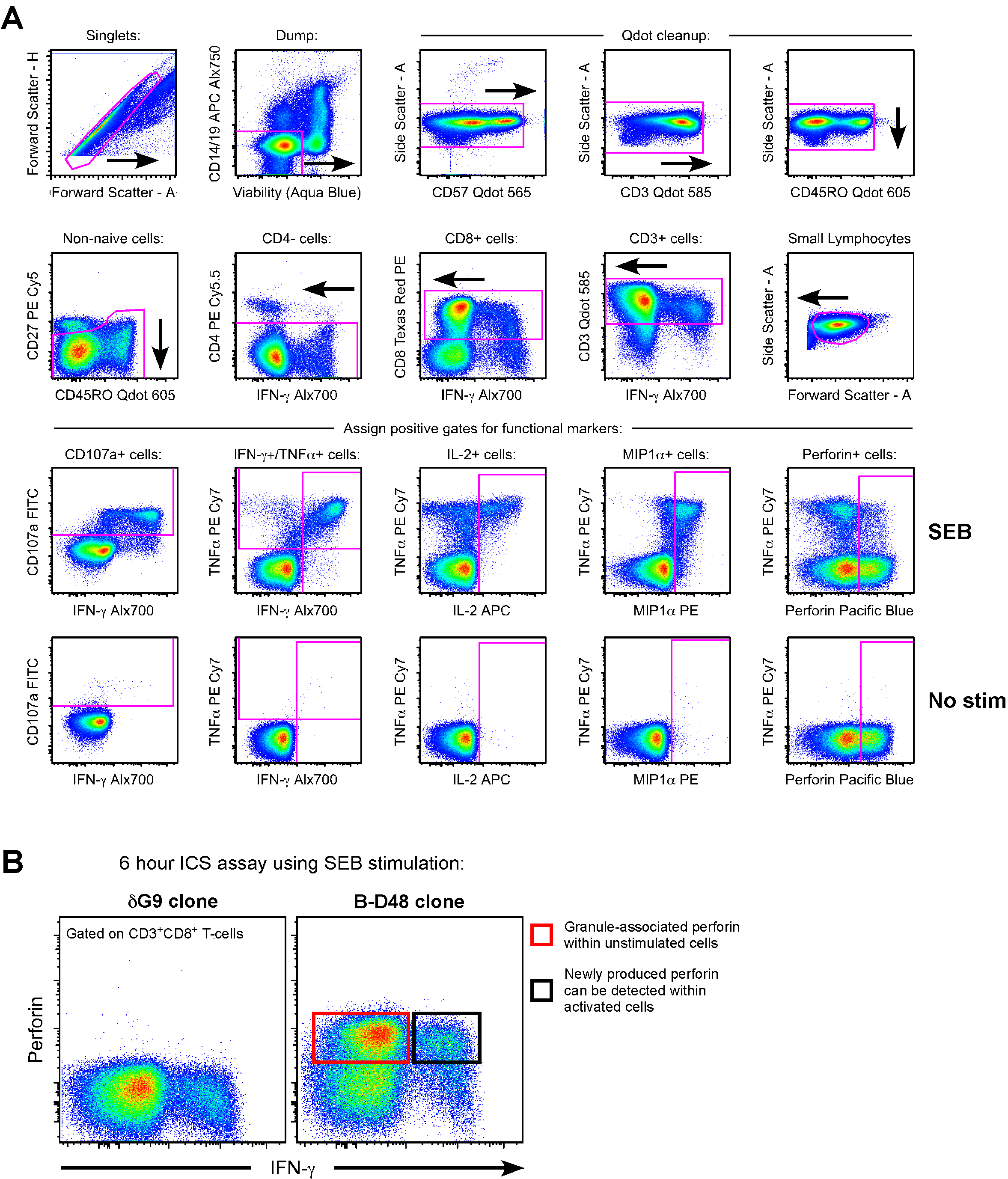

Supplement: Figure S1 — Perforin upregulation can be detected using a polychromatic flow cytometric staining panel. (A) The gating strategy from a representative subject: PBMC from an EC were stimulated with SEB for six hours and then stained for six CD8+ T-cell functions (perforin, CD107a, IFN-γ, IL-2, TNFα, and MIP1α) along with lineage (CD14, CD19, CD3, CD4, CD8) and memory (CD27, CD45RO, and CD57) markers. The no stimulation control is also shown. (B) PBMC were stimulated with SEB for six hours in the presence of BFA and monensin. Perforin was stained either using the δG9 (left) or B-D48 (right) antibody clones. The red box denotes granule-associated perforin within the population of CD8+ T-cells that did not respond to SEB stimulation (i.e. resting CD8+ T-cells not producing IFN-γ). The black box denotes a population of CD8+ T-cells expressing both perforin and IFN-γ that can be detected using the B-D48 clone in a conventional ICS assay. (0.60 MB TIF) [file ppat.1000917.s001.tif]

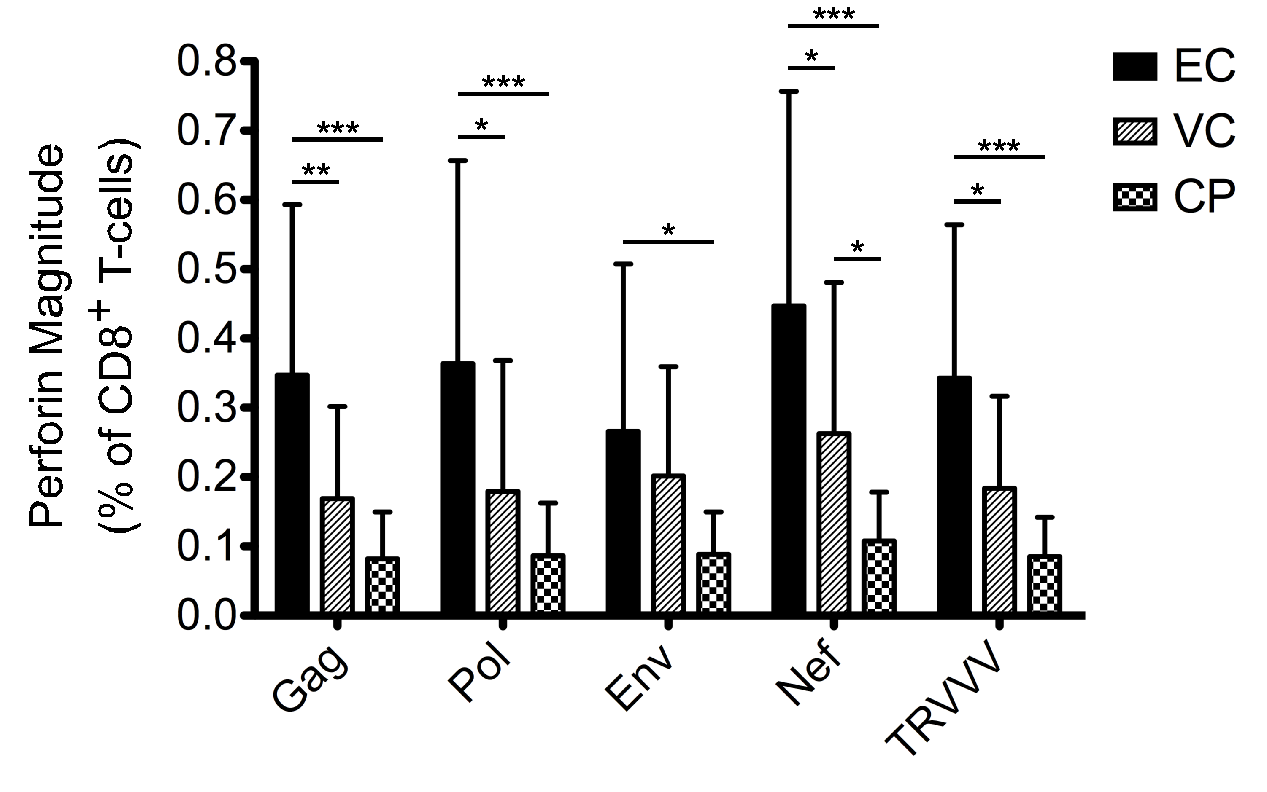

Supplement: Figure S2 — EC demonstrate higher perforin magnitude than CP. In addition to the relative contribution of perforin to the HIV-specific CD8+ T-cell response, EC also demonstrate higher perforin magnitude. Total perforin production induced by each HIV antigen pool is represented as the frequency of CD8+ T-cells (excluding naïve cells). One-way ANOVA tests (nonparametric; Kruskal-Wallis test) were performed followed by a Dunns test for multiple comparisons. * denotes a p value < 0.05, ** denotes a p value < 0.01, and *** denotes a p value < 0.001. All bars represent the mean and error bars indicate the standard deviation. (0.08 MB TIF) [file ppat.1000917.s002.tif]

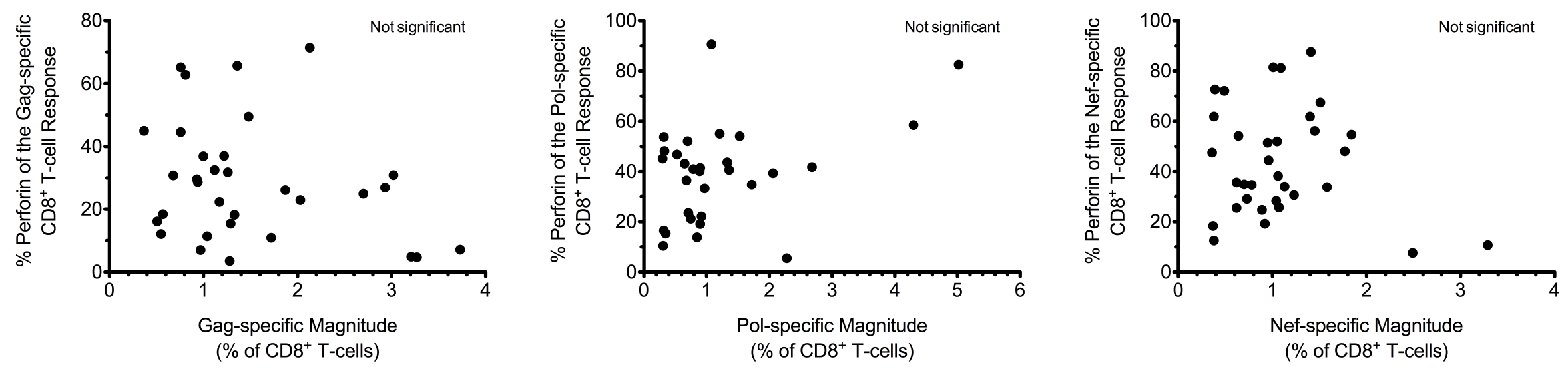

Supplement: Figure S3 — There is no association between HIV response magnitude and corresponding perforin expression. The Gag-, Pol-, and Nef-specific response magnitude (as the frequency of CD8+ T-cells; excluding naïve cells) is plotted against the corresponding proportion of perforin expression for each CD8+ T-cell response among all EC subjects. Spearman correlation tests (nonparametric; two-tailed) revealed no statistically significant relationship. (0.16 MB TIF) [file ppat.1000917.s003.tif]

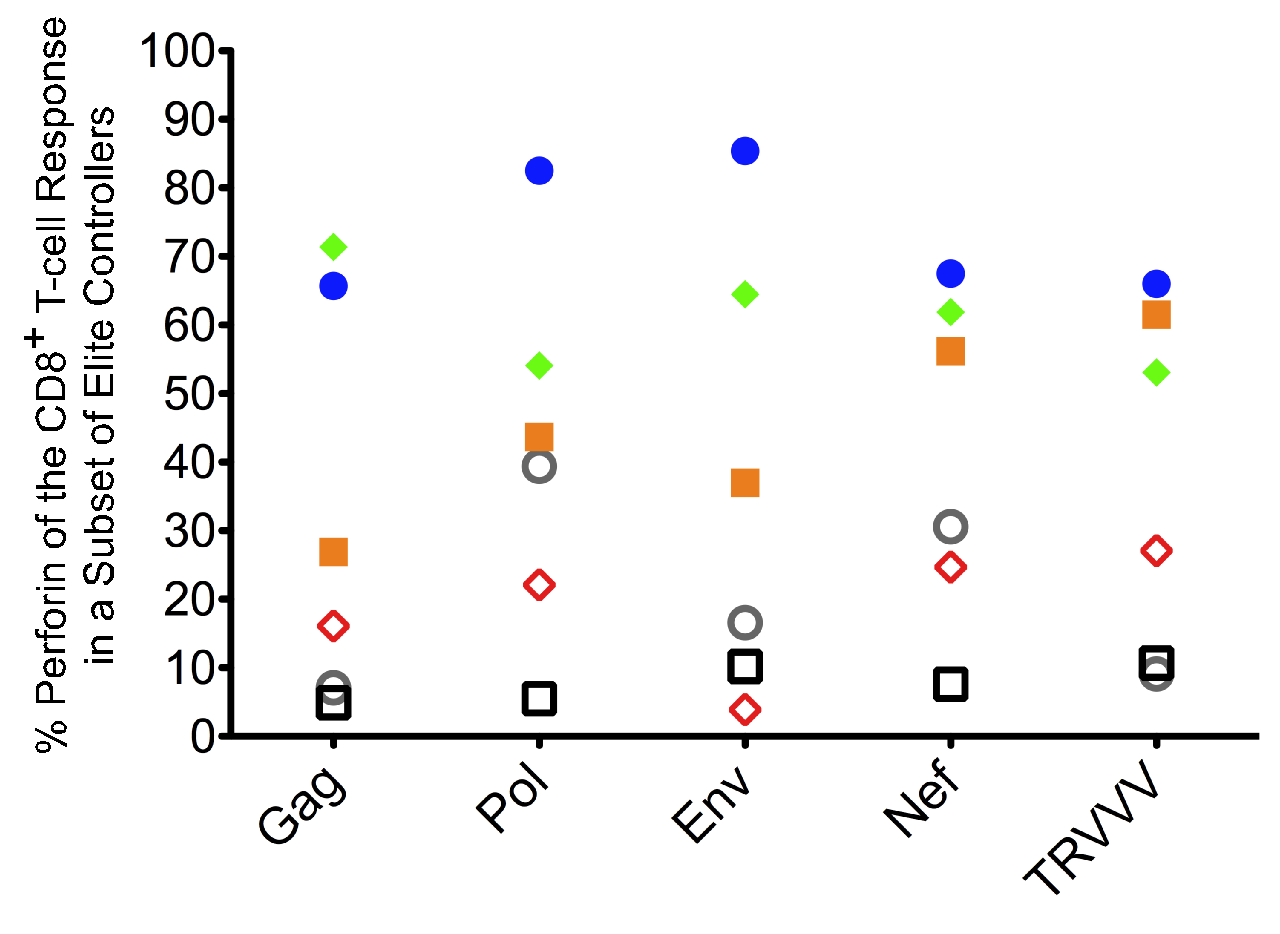

Supplement: Figure S4 — EC demonstrate some variability in HIV-specific perforin expression. The relative contribution of perforin for the CD8+ T-cell response to each HIV antigen pool is shown for a selected subset of EC. Each symbol represents a different EC subject, and symbols of the same color represent responses from the same individual. These subjects were chosen partly because they mounted a positive response to all five HIV peptide pools. (0.06 MB TIF) [file ppat.1000917.s004.tif]

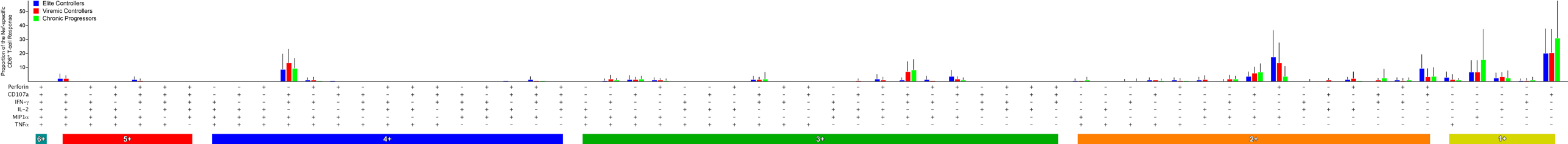

Supplement: Figure S5 — Breakdown of the average Nef-specific response from EC, VC, and CP into all 64 possible functional permutations. The entire response was broken down into the contribution of each functional combination for the average Nef-specific CD8+ T-cell response. Note that two functional permutations are ignored in the analysis: perforin single positive and all negative. All bars represent the mean and error bars indicate the standard deviation. (0.14 MB TIF) [file ppat.1000917.s005.tif]

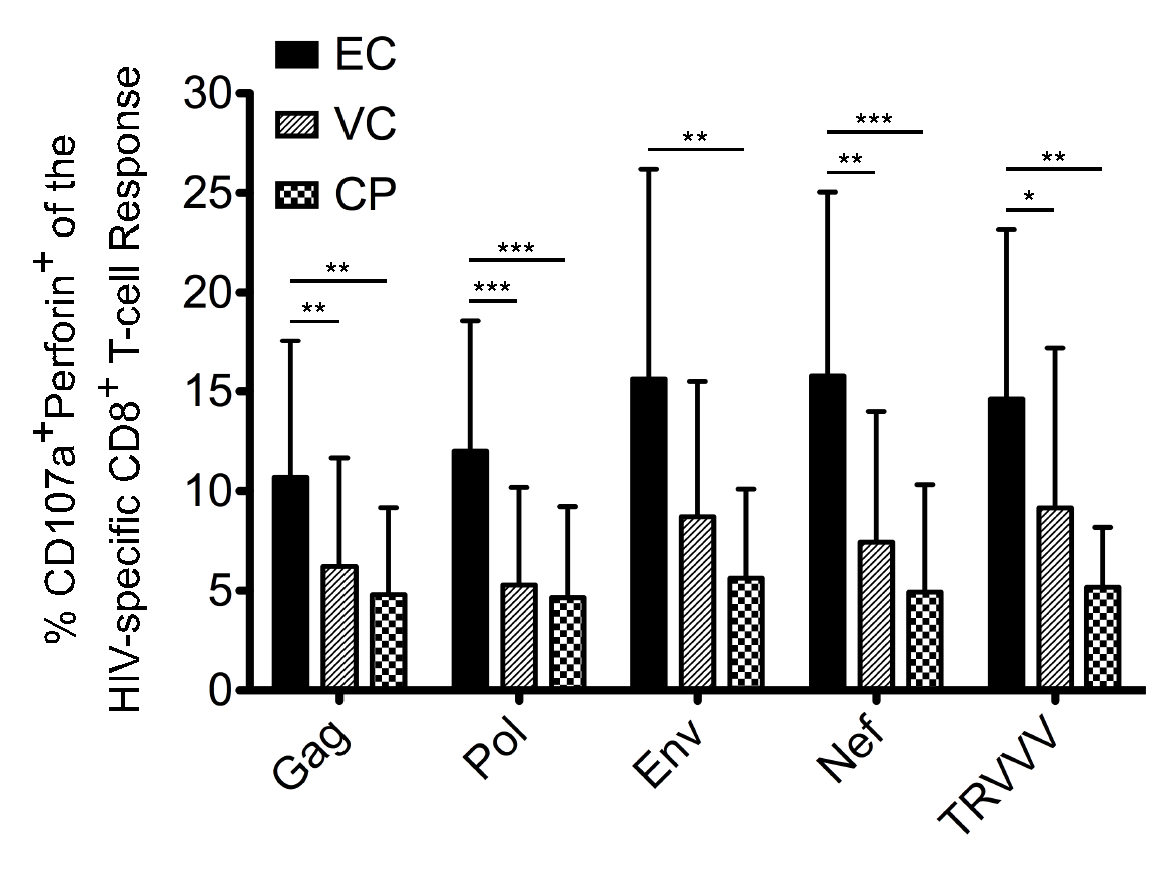

Supplement: Figure S6 — EC have an increased capacity for de novo perforin synthesis. The proportion of the CD8+ T-cell response comprised of every CD107a+perforin+ functional subset was calculated for all HIV antigens in EC, VC, and CP. One-way ANOVA tests (nonparametric; Kruskal-Wallis test) were performed followed by a Dunns test for multiple comparisons. * denotes a p value < 0.05, ** denotes a p value < 0.01, and *** denotes a p value < 0.001. All bars represent the mean and error bars indicate the standard deviation. (0.10 MB TIF) [file ppat.1000917.s006.tif]

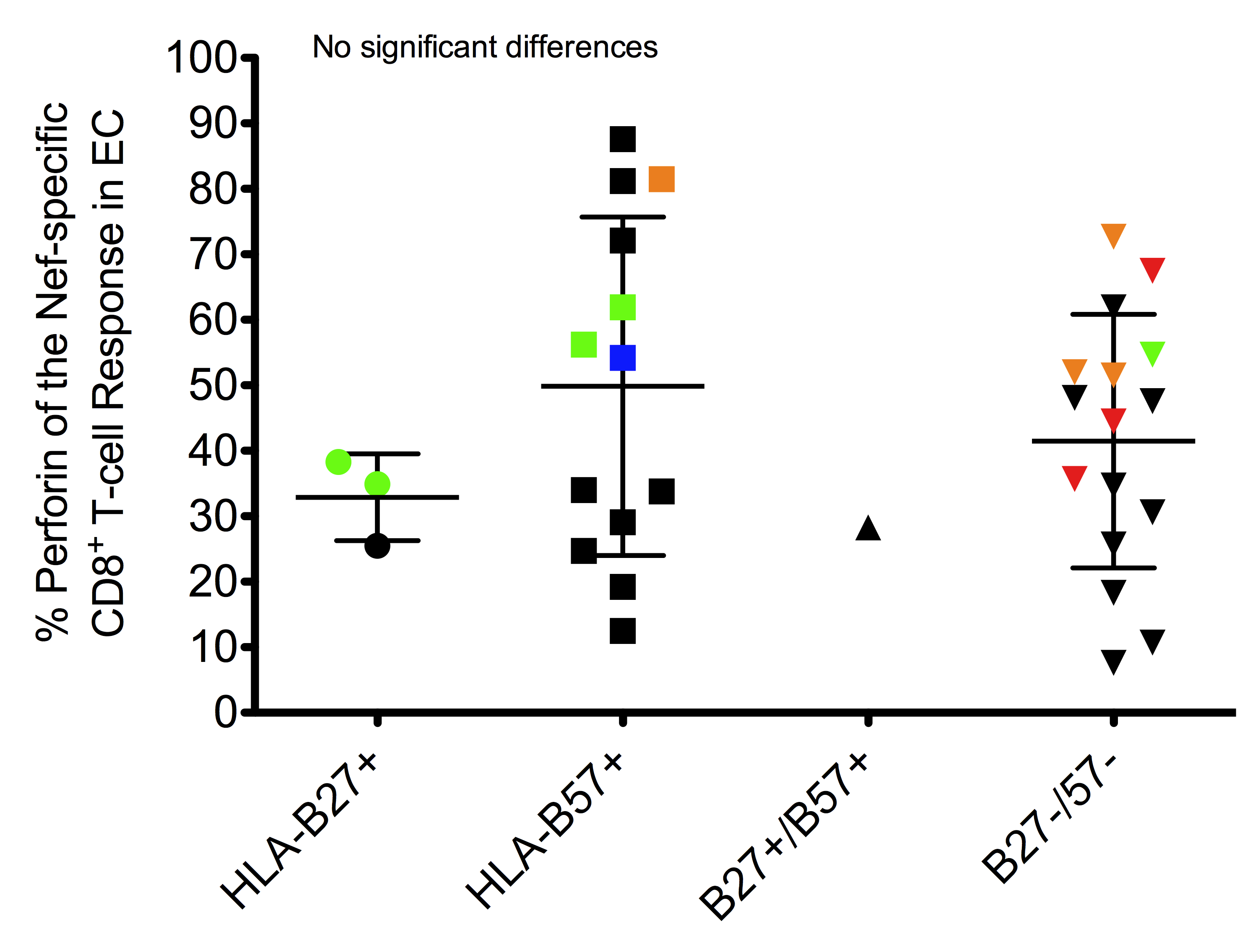

Supplement: Figure S7 — Perforin expression is not restricted to the presence of protective HLA-B alleles. EC were stratified based on the expression of HLA-B alleles previously shown to be associated with improved clinical outcomes. The relative amount of perforin expression is shown for the Nef-specific CD8+ T-cell responses among all EC. Each symbol represents an individual study subject. Some of the symbols are colored to denote the presence of another protective HLA-B allele: blue, HLA-B13; green, HLA-B15; orange, HLA-B51; red, HLA-B58. No statistically significant differences were found between the groups using a one-way ANOVA test (nonparametric; Kruskal-Wallis) followed by a Dunns test for multiple comparisons. The error bars represent the mean and standard deviation. (0.21 MB TIF) [file ppat.1000917.s007.tif]

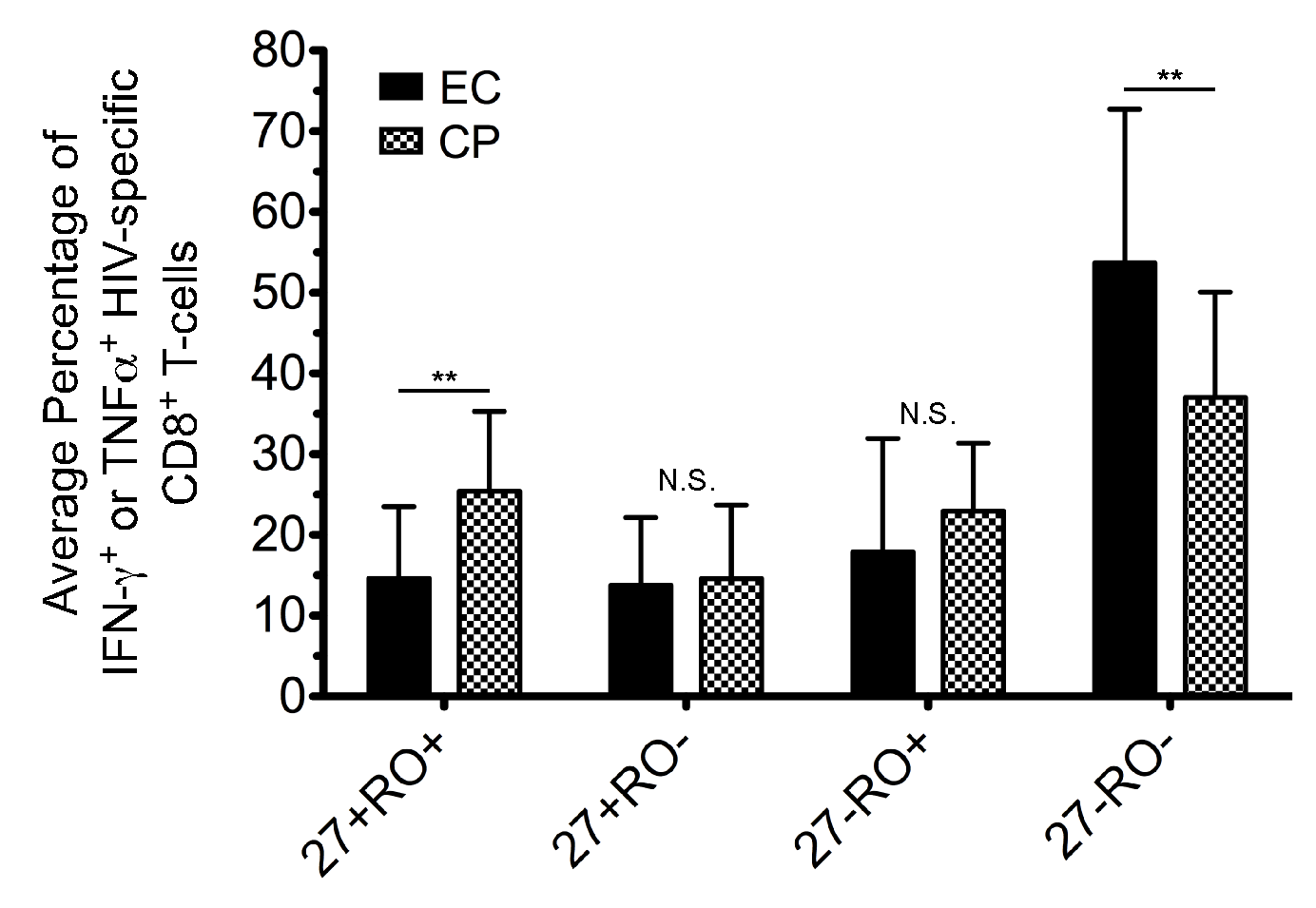

Supplement: Figure S8 — EC display an expansion of CD27-CD45RO- effector HIV-specific CD8+ T-cells. The memory phenotype, based on the surface expression of CD27 and CD45RO, was determined for the average HIV-specific CD8+ T-cell response, as defined by the production of IFN-γ or TNFα, among EC and CP. Mann-Whitney tests (nonparametric; two-tailed) were performed for each phenotypic combination. ** denotes a p value < 0.01. All bars represent the mean and error bars indicate the standard deviation. (0.09 MB TIF) [file ppat.1000917.s008.tif]

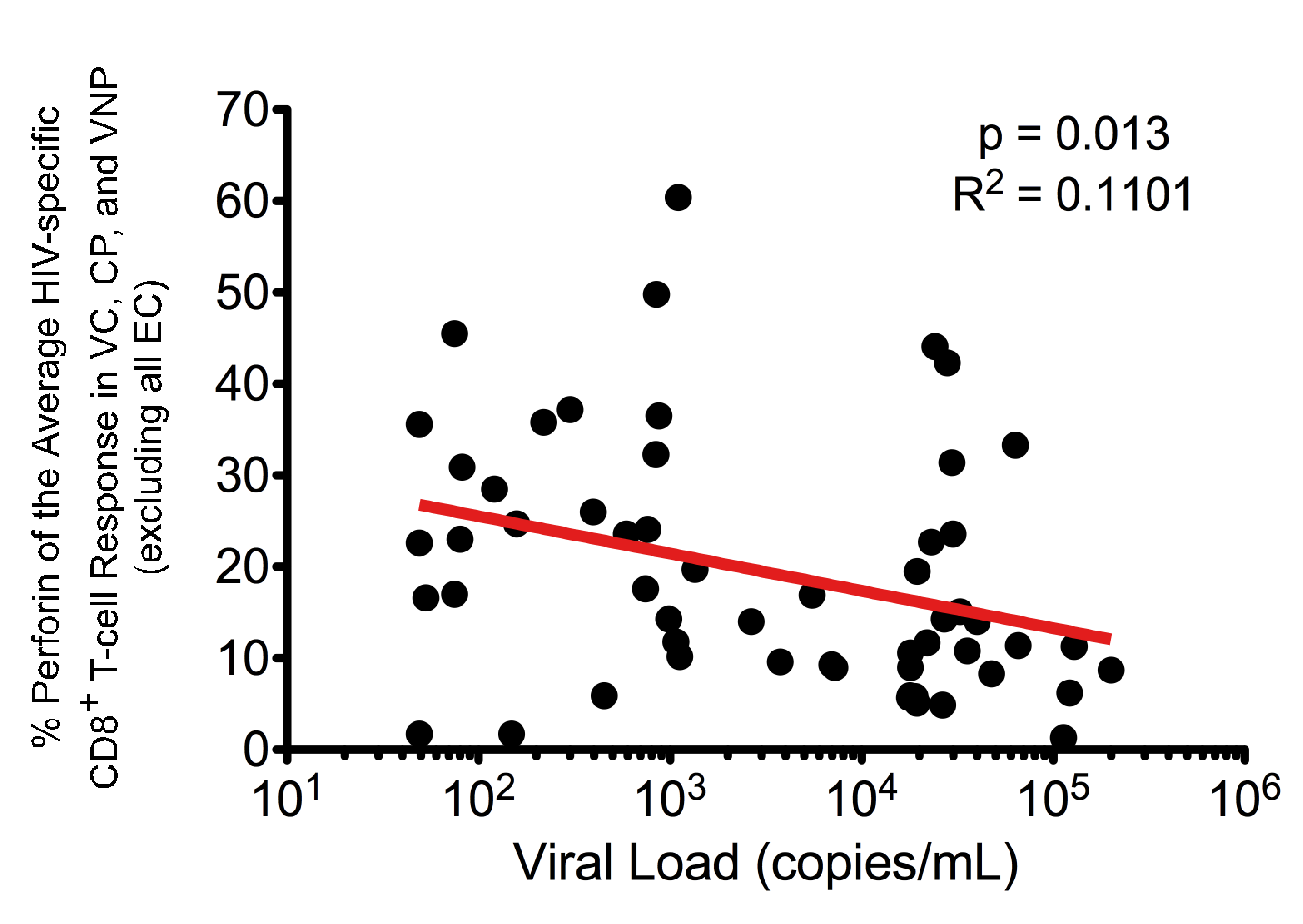

Supplement: Figure S9 — Negative correlation between HIV-specific perforin expression and viral load when considering only VC, CP, and VNP subjects. The average percentage of HIV-specific perforin expression from CD8+ T-cells within each subject was plotted against the HIV viral load among all subjects excluding EC. The most proximal viral load measurement to the time point of the PBMC sample was used in the analysis. Spearman correlation tests (nonparametric; two-tailed) were performed to determine statistical significance. (0.09 MB TIF) [file ppat.1000917.s009.tif]

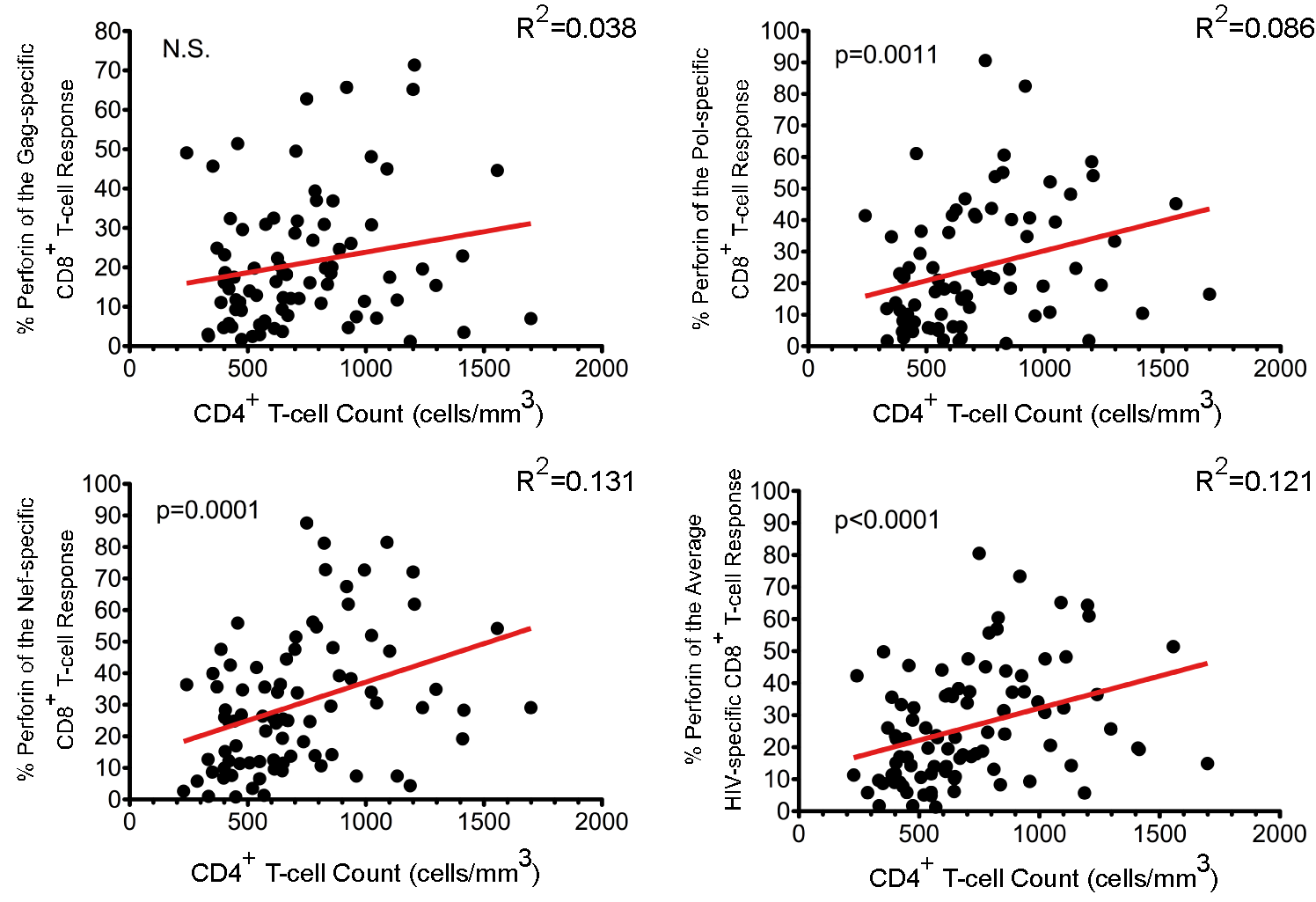

Supplement: Figure S10 — Positive correlation between HIV-specific perforin expression and peripheral blood CD4+ T-cell counts. The percentage of Gag-, Pol-, and Nef-specific perforin expression within each subject was plotted against CD4+ T-cell counts. The average percentage of HIV-specific perforin expression within each individual was also plotted against CD4+ T-cell counts. The most proximal CD4+ T-cell count to the time point of the PBMC sample was used in the analysis. Spearman correlation tests (nonparametric; two-tailed) were performed to determine statistical significance. (0.17 MB TIF) [file ppat.1000917.s010.tif]

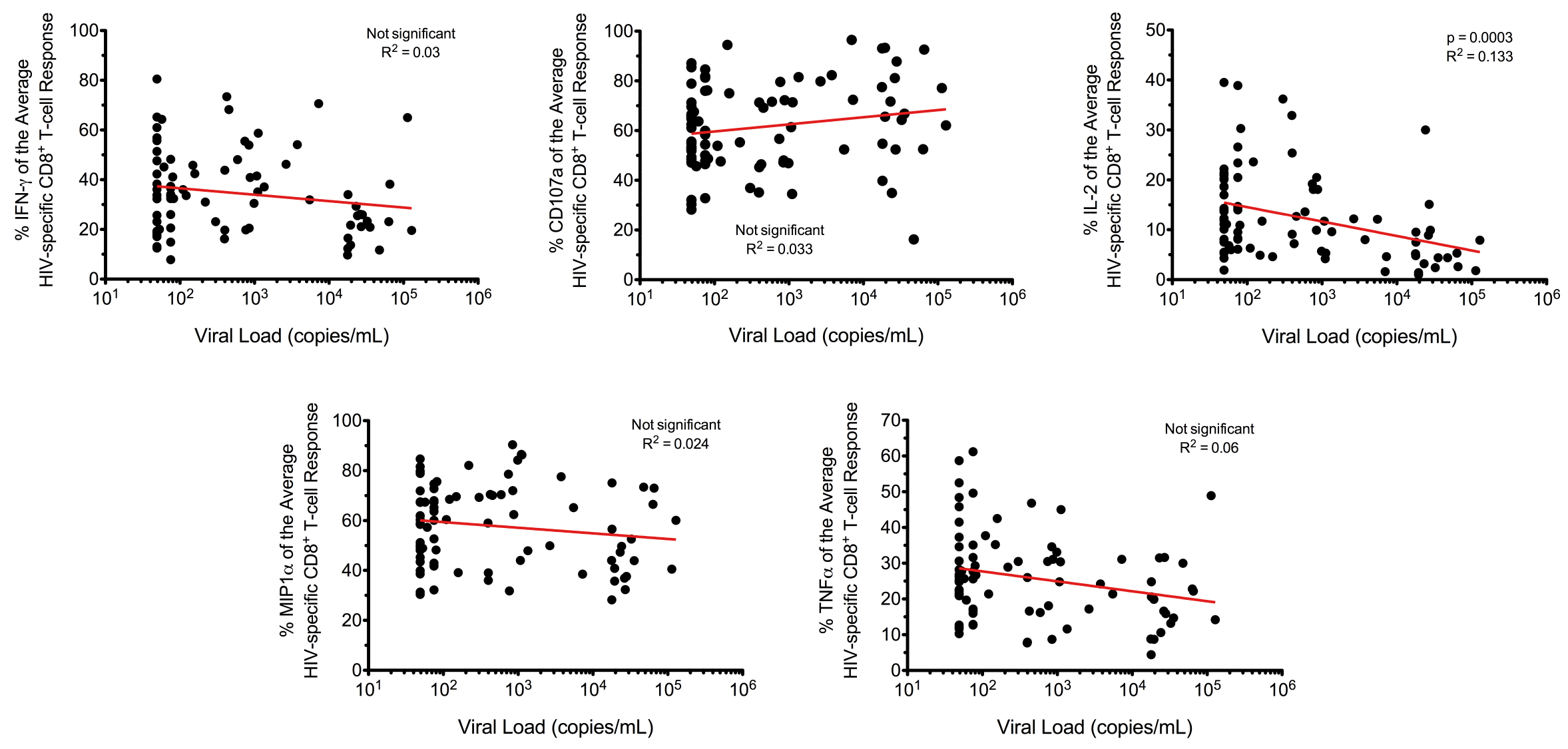

Supplement: Figure S11 — Not all functional parameters are correlated with control of HIV replication. The proportion of each measured functional parameter (except perforin) comprising the average HIV-specific CD8+ T-cell response in each subject was plotted against the HIV viral load from each respective subject. The most proximal viral load measurement to the time point of the PBMC sample was used in the analysis. Spearman correlation tests (nonparametric; two-tailed) were performed to determine statistical significance. (0.31 MB TIF) [file ppat.1000917.s011.tif]

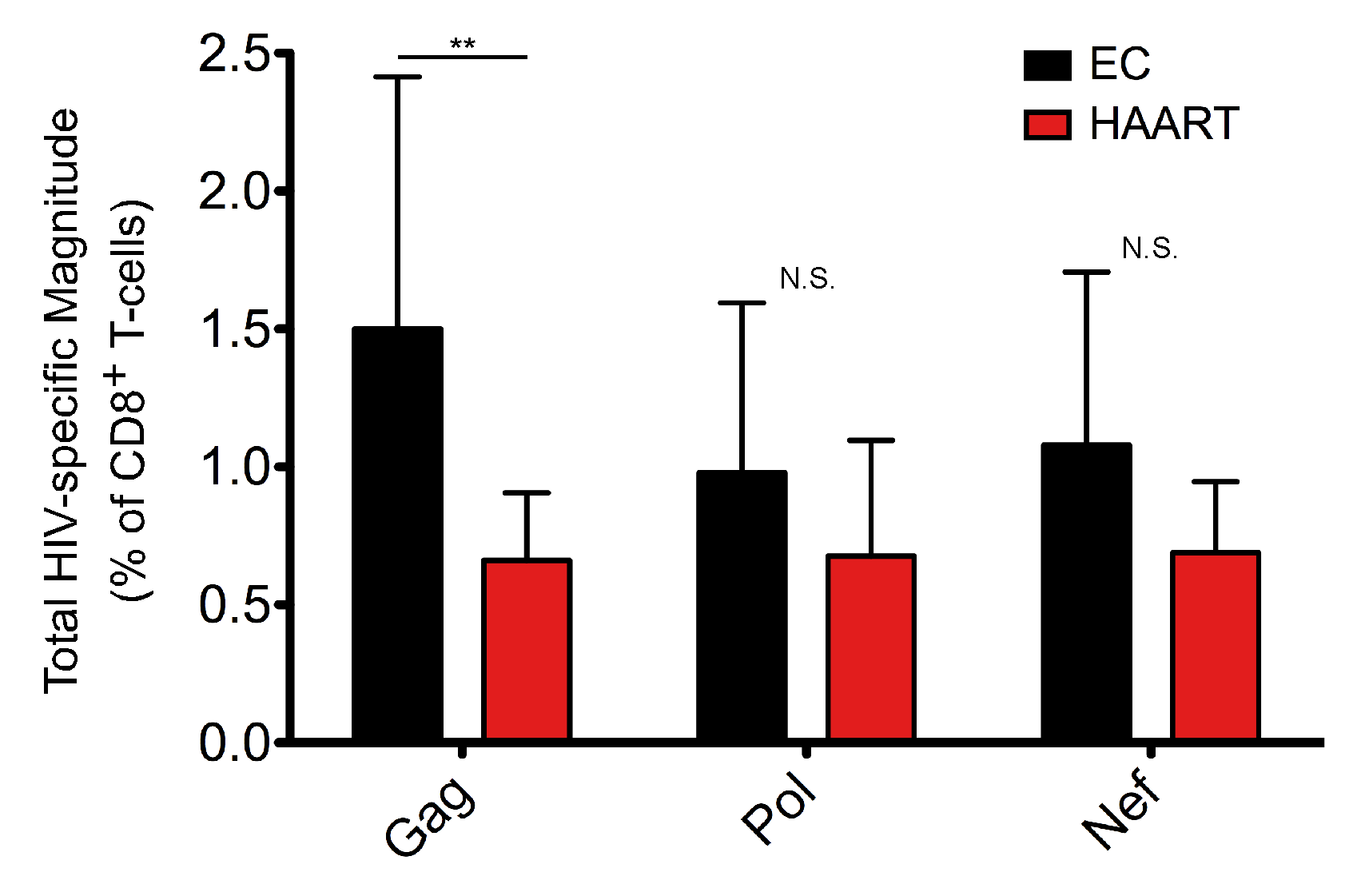

Supplement: Figure S12 — HIV-specific CD8+ T-cell response magnitude is slightly higher in EC than HAART-treated individuals. The CD8+ T-cell response magnitude to Gag, Pol, and Nef peptide pools was calculated for EC and HAART-treated subjects and plotted as percent of CD8+ T-cells (excluding naïve cells). The total magnitude was calculated by summing across all functional combinations. Mann-Whitney tests (nonparametric; two-tailed) were performed for each HIV antigen. ** denotes a p value < 0.01. All bars represent the mean and error bars indicate the standard deviation. (0.06 MB TIF) [file ppat.1000917.s012.tif]

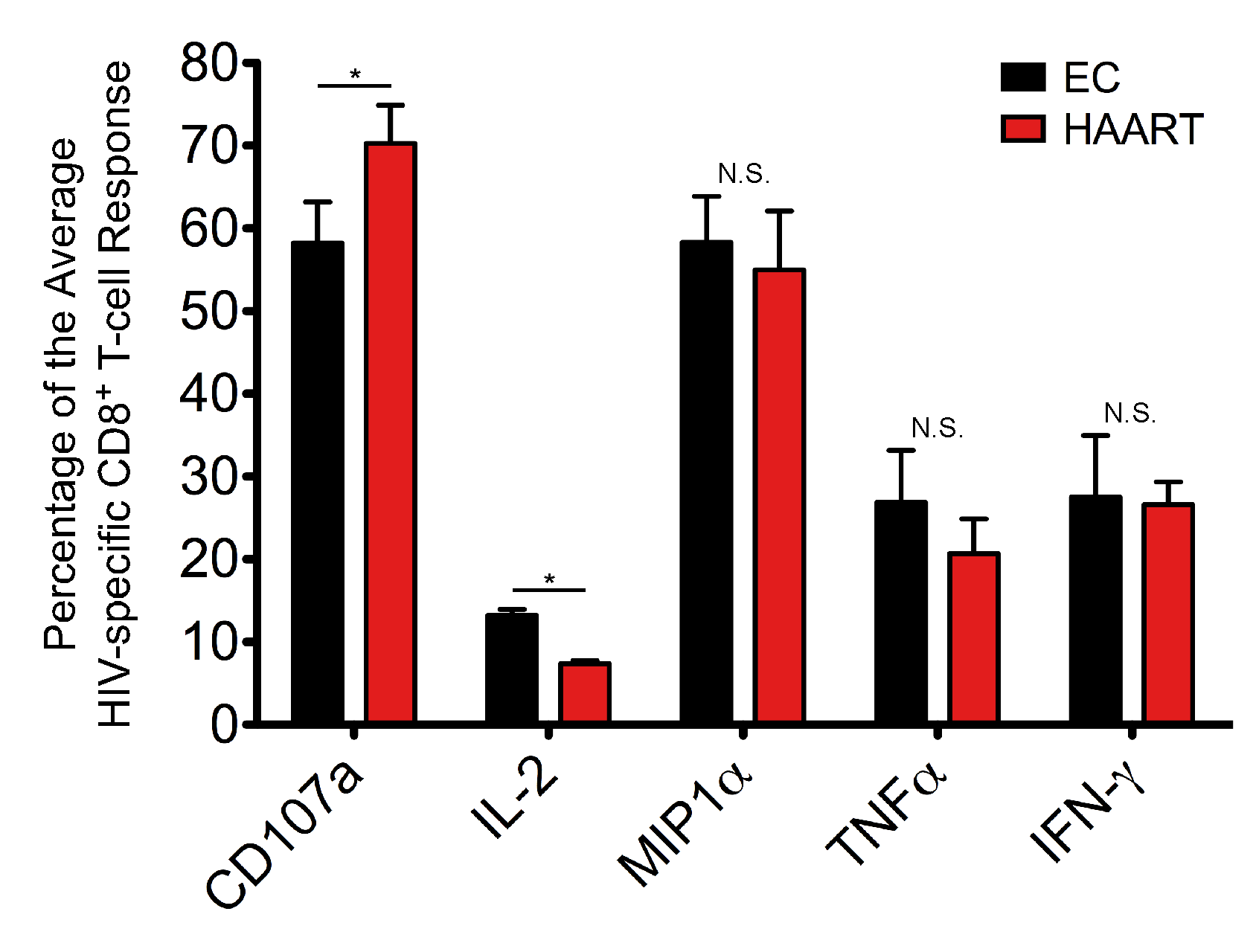

Supplement: Figure S13 — The HIV-specific CD8+ T-cell response between EC and HAART-treated subjects does not vary greatly in degranulation, cytokine production, or chemokine expression. The proportion of the average HIV-specific CD8+ T-cell response comprised of each single functional parameter (except perforin) is shown among EC and HAART-treated subjects. Mann-Whitney tests (nonparametric; two-tailed) were performed for each functional parameter. * denotes a p value < 0.05. All bars represent the mean and error bars indicate the standard deviation. (0.09 MB TIF) [file ppat.1000917.s013.tif]

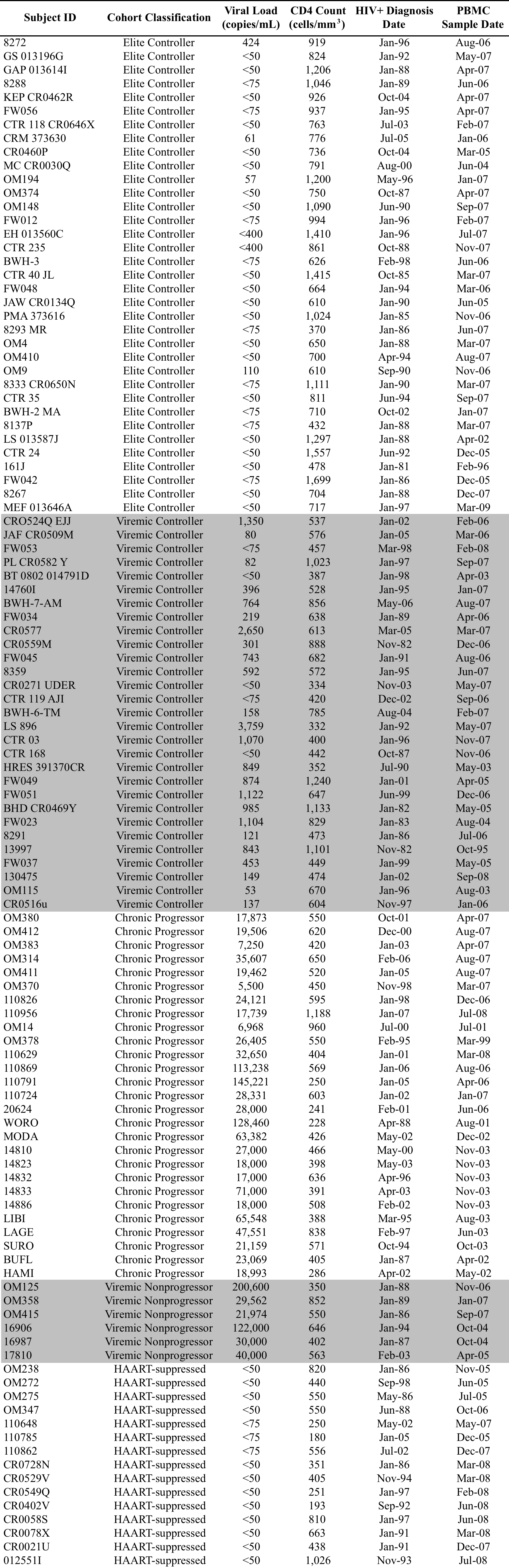

Supplement: Table S1 — Complete study cohort with relevant clinical parameters. (1.50 MB TIF) [file ppat.1000917.s014.tif]
